# Tetrahydroquinolinone derivatives exert antiproliferative effect on lung cancer cells through apoptosis induction

**DOI:** 10.1038/s41598-022-23640-9

**Published:** 2022-11-09

**Authors:** Małgorzata Ryczkowska, Natalia Maciejewska, Mateusz Olszewski, Milena Witkowska, Sławomir Makowiec

**Affiliations:** 1 Department of Organic Chemistry, Faculty of Chemistry, Gdansk University of Technology, Narutowicza 11/12, 80-233 Gdansk, Poland; 2 Department of Pharmaceutical Technology and Biochemistry, Faculty of Chemistry, Gdansk University of Technology, Narutowicza 11/12, 80-233 Gdansk, Poland

**Keywords:** Lung cancer, Synthetic chemistry methodology, Lung cancer

## Abstract

The anticancer properties of quinolones is a topic of interest among researchers in the scientific world. Because these compounds do not cause side effects, unlike the commonly used cytostatics, they are considered a promising source of new anticancer drugs. In this work, we designed a brief synthetic pathway and obtained a series of novel 8-phenyltetrahydroquinolinone derivatives functionalized with benzyl-type moieties at position 3. The compounds were synthesized via classical reactions such as nucleophilic substitution, solvent lysis, and condensation. Biological evaluation revealed that 3-(1-naphthylmethyl)-4-phenyl-5,6,7,8-tetrahydro-1H-quinolin-2-one (**4a**) exhibited potent cytotoxicity toward colon (HTC-116) and lung (A549) cancer cell lines. Analysis of the mechanism of action of compounds showed that compound **4a** induced cell cycle arrest at the G_2_/M phase, leading to apoptotic cell death via intrinsic and extrinsic pathways. Taken together, the findings of the study suggest that tetrahydroquinolinone derivatives bearing a carbonyl group at position 2 could be potential lead compounds to develop anticancer agents for the treatment of lung cancers.

## Introduction

Apoptosis or programmed cell death is a basic physiological process that plays a key role in the maintenance of tissue homeostasis. It is genetically regulated as a normal physiological response to many stimuli and is associated with other processes such as aging and embryogenesis. Disorders in apoptosis mechanisms can lead to various diseases, such as cancers. The disability of cancerous cells to keep up the balance between proliferation and death results in the development of abnormal tissue and formation of solid tumors^1,2^. Apoptosis is a double-track mechanism that occurs via extrinsic and intrinsic pathways. The extrinsic pathway involves transmembrane death receptor-mediated interactions, whereas the intrinsic pathway is mediated by mitochondria and starts with the binding of BAX/BAK protein to the mitochondrial membrane leading to the release of cytochrome c^3^. Both pathways converge at the same point—caspases-regulated execution. The majority of cytostatic drugs used in anticancer therapy cause burdensome side effects in patients; therefore, there is a constant search for novel chemical compounds that are safe. Quinolones are a family of compounds characterized by antibacterial properties. However, research indicates that some of them exhibit potential anticancer properties, especially apoptosis activation. It has been proven that ciprofloxacin, which is the most active fluoroquinolone, can activate apoptosis in breast, bladder, and prostate cancers, colorectal carcinoma, and melanoma. At the molecular level, this chemotherapeutic causes an adverse increase in the concentration of BAX protein which results in differences in the BAX:BCL-2 ratio. It also enhances the expression of p53 protein and activation of caspases^4–8^. Another fluoroquinolone, enoxacin, also works by activating apoptosis. It has been shown that programmed cell death is induced in prostate cancer by a significant increase in CASP3 mRNA and cleaved PARP expression as well as mitochondrial depolarization^9^. Levofloxacin, which is also a fluoroquinolone, induces apoptosis in breast and lung cancer through a caspase-dependent pathway and mitochondrial disfunction^10^. Obviously, several well-known chemotherapeutic agents from the quinolone family display anticancer properties^11^. One of the newer quinolones, voreloxin, which is currently in clinical trials for the treatment of acute myeloid leukemia, shows a high affinity to eukaryotic type II topoisomerase and induces apoptosis through double-strand DNA breaks. Because the stable quinolone core is characterized by a favorable toxicity profile, voreloxin does not generate reactive oxygen species and free radicals unlike the widely used anthracyclines. Hence, this compound is much less harmful to the off-target tissues compared to the commonly used chemotherapeutic drugs^12,13^.

**Figure 1:**
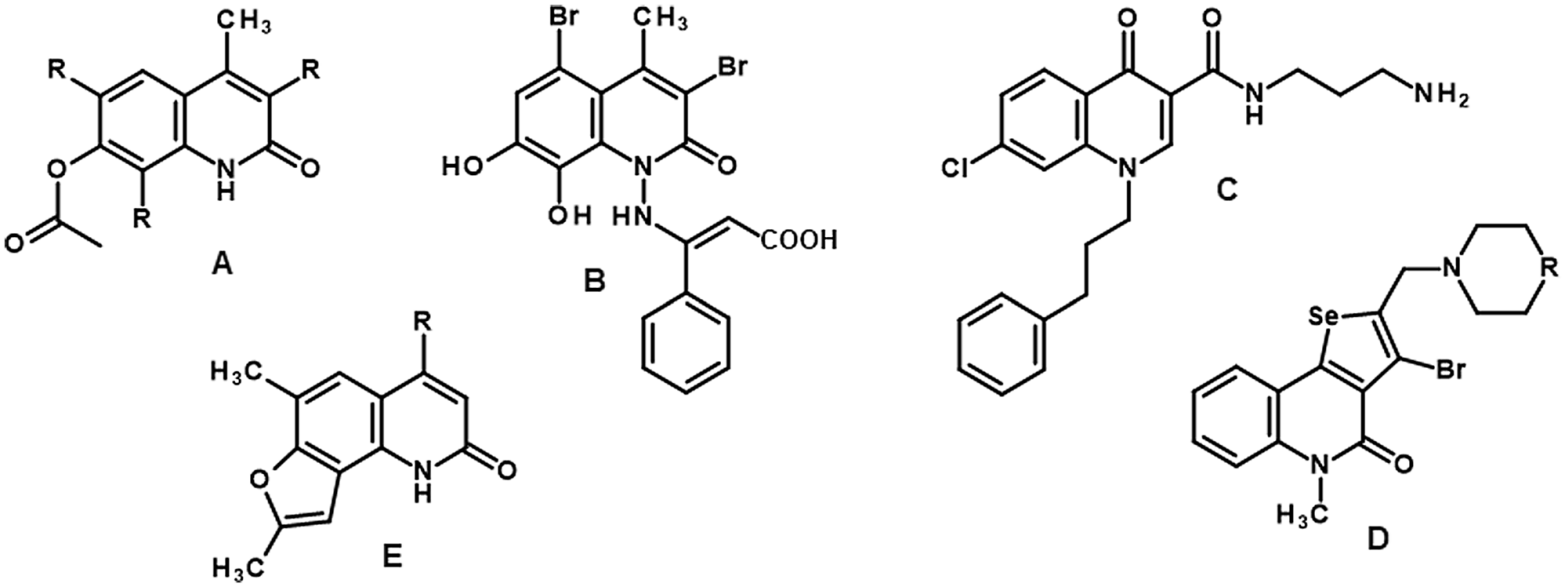
Examples of modified quinolinone cores that induced apoptosis in various cancer cells.

Over the past few years, researchers have proposed several interesting short synthesis pathways for the formation of various quinolinone cores. A.M.Y. Moustafa and S.B. Bakare described the synthesis of new 7-hydroxy quinolinone derivatives based on parent 7-hydroxy-4-methyl-2-oxo-1-amino quinolone. A series of new chemical compounds containing different substituents at positions 3–8 were assessed for their biological activity against breast cancer. It was observed that the most active compound **A** (Fig. 1) increased the level of active caspase-3, leading to the induction of apoptosis via mitochondrial pathway^14^. An interesting study demonstrated the synthesis of hybrid molecules containing cinnamic acid and 1-amino-4-methyl-2-quinolinone derivatives. The final products were synthesized via pyrogallol and ethyl acetoacetate condensation, resulting in the formation of 7,8-dihydroxy-4-methyl-coumarin which was then treated with hydrazine in pyridine to obtain a desirable 2-quinolinone core. Biological studies showed that compound **B**, which was qualified for further assessment, caused an increase in apoptotic cell fraction in the HCT-116 cell line^15^. Zhou P. et al*.* also proposed a few-step synthetic pathway for novel 4(1H)-quinoline derivatives based on the lead compound 7-chloro-4(1H)-quinolone characterized by poor solubility. In this study, five novel, functionalized chemical compounds were obtained via a previously described procedure to improve the solubility of final products. All these compounds exhibited antitumor activity; however, structure **C** also displayed proapoptotic properties and increased the level of Bak protein, inducing cell cycle arrest at the G2/M phase via a p53-dependent pathway in the HepG2 cell line^16^. Another study described the brief synthesis of ring-fused quinolinones. P. Arsenyan et al*.* proposed simple methods for the synthesis of substituted selenopheno[2,3-c] and selenopheno[3,2-c] quinolinones **D**^17^. Biological evaluation of compounds showed that these derivatives induced apoptosis via an intrinsic pathway in human breast adenocarcinoma, estrogen-positive (MCF-7) tumor cell line^18^. A. Chilin et al*.* reported a novel, more convenient pathway for synthesizing 1,6,8-trimethylfuro[2,3-h]quinolin-2-one derivatives **E**, which induced programmed cell death through mitochondrial depolarization. The synthesized compounds exerted a cytotoxic effect in Jurkat cells in the dark, which was dependent on ADH-catalyzed oxidation^19^. Finally, new tetrahydroquinoline derivatives synthesized in our previous work demonstrated potent anticancer activity on human colon cancer cells (HTC-116) and nonsmall cell lung cancer cells (A549). The literature review and experience derived from this project inspired us to propose and evaluate a short synthetic route for the synthesis of new tetrahydroquinoline derivatives modified with benzyl-type substituents at position 3^20^.

### Chemistry

#### Synthesis of tetrahydroquinoline derivatives modified at position 3

**Figure 2:**
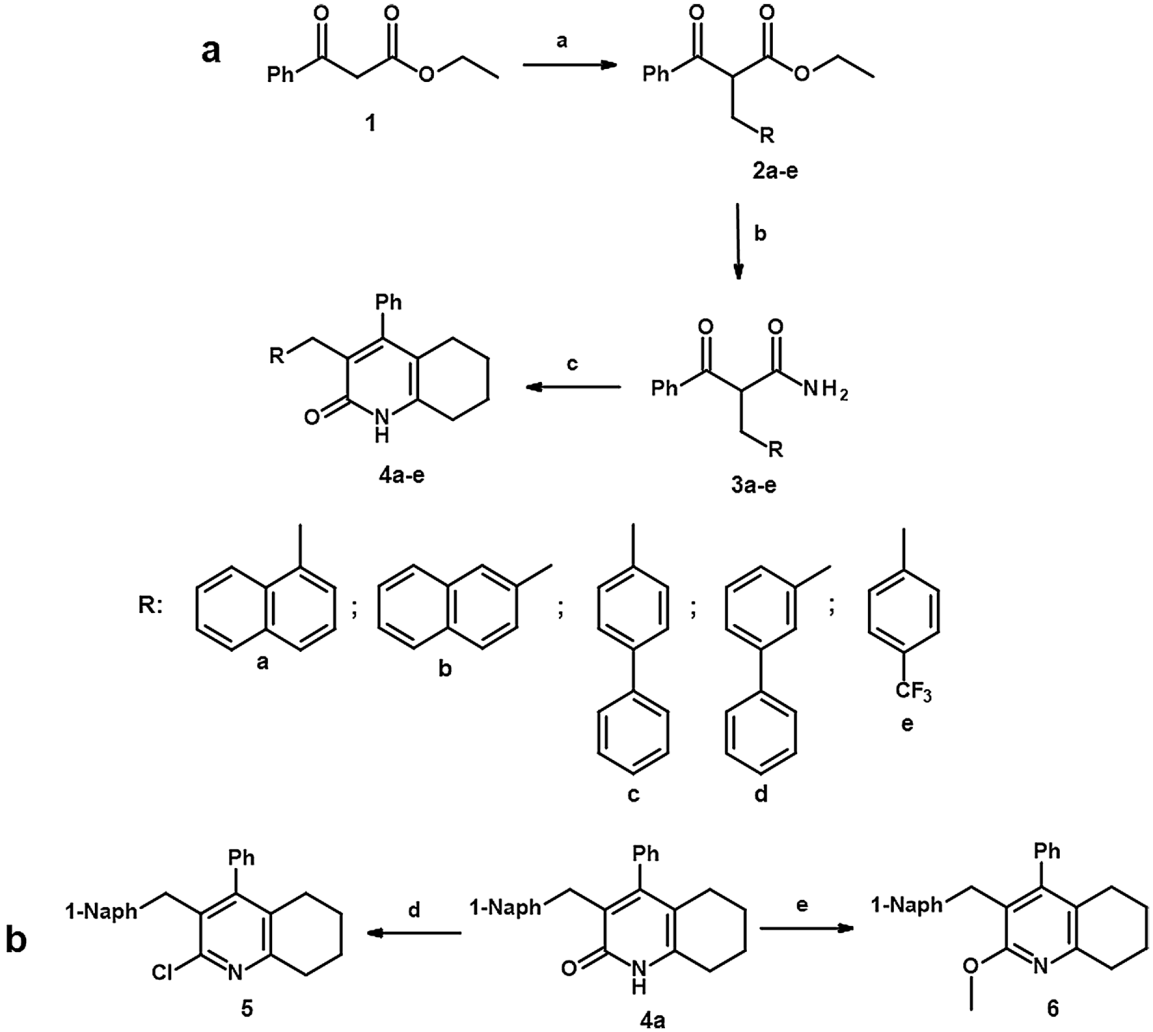
Synthetic pathways for the preparation of new tetrahydroquinolinone derivatives: (**a**) RCH_2_X, K_2_CO_3_/DMF, 60 °C, 5 h; (**b**) 24% NH_3_ *aq*, 50 °C, 24–72 h; (**c**) TsOH/toluene, reflux, 12 h; (**d**) PhP(O)Cl_2_, 160 °C, 16 h; (**e**) Ag_2_CO_3_, CH_3_I/CHCl_3_, RT, 12 h.

We synthesized the new tetrahydroquinoline derivatives through the following three-step pathway (Fig. 2a). The starting compound was ethyl benzoylacetate (**1**). In the first step, ethyl benzoylacetate and a variety of benzyl-type halides were subjected to the S_N_2 reaction. The reaction was carried out in dimethylformamide (DMF) in the presence of K_2_CO_3_. Then, the resulting benzyl-type derivatives of ethyl benzoylacetate (**2a–e**) were subjected to ammonolysis in 24% ammonia water solution. The corresponding benzoylopropanamides (**3a–e**) were condensed with cyclohexanone in the presence of TsOH and anhydrous MgSO_4_ to obtain desirable tetrahydroquinolinones (**4a–e**) modified with benzyl-type substituents at position 3^21^. After biological evaluation, the most active 3-(1-naphthylmethyl)-4-phenyl-5,6,7,8-tetrahydro-1H-quinolin-2-one (**4a**) was converted into chlor^22^ and methoxy^23^ derivatives (**5**, **6**) (Fig. 2b). It is worth mentioning that in some cases tautomers and rotamers were observed, which is described in the supplementary information.

### Biology evaluation

#### Compound *4a* significantly inhibits the viability of human nonsmall cell lung cancer and colon cancer cells with minimal toxicity to nonmalignant human kidney cells

**Figure 3:**
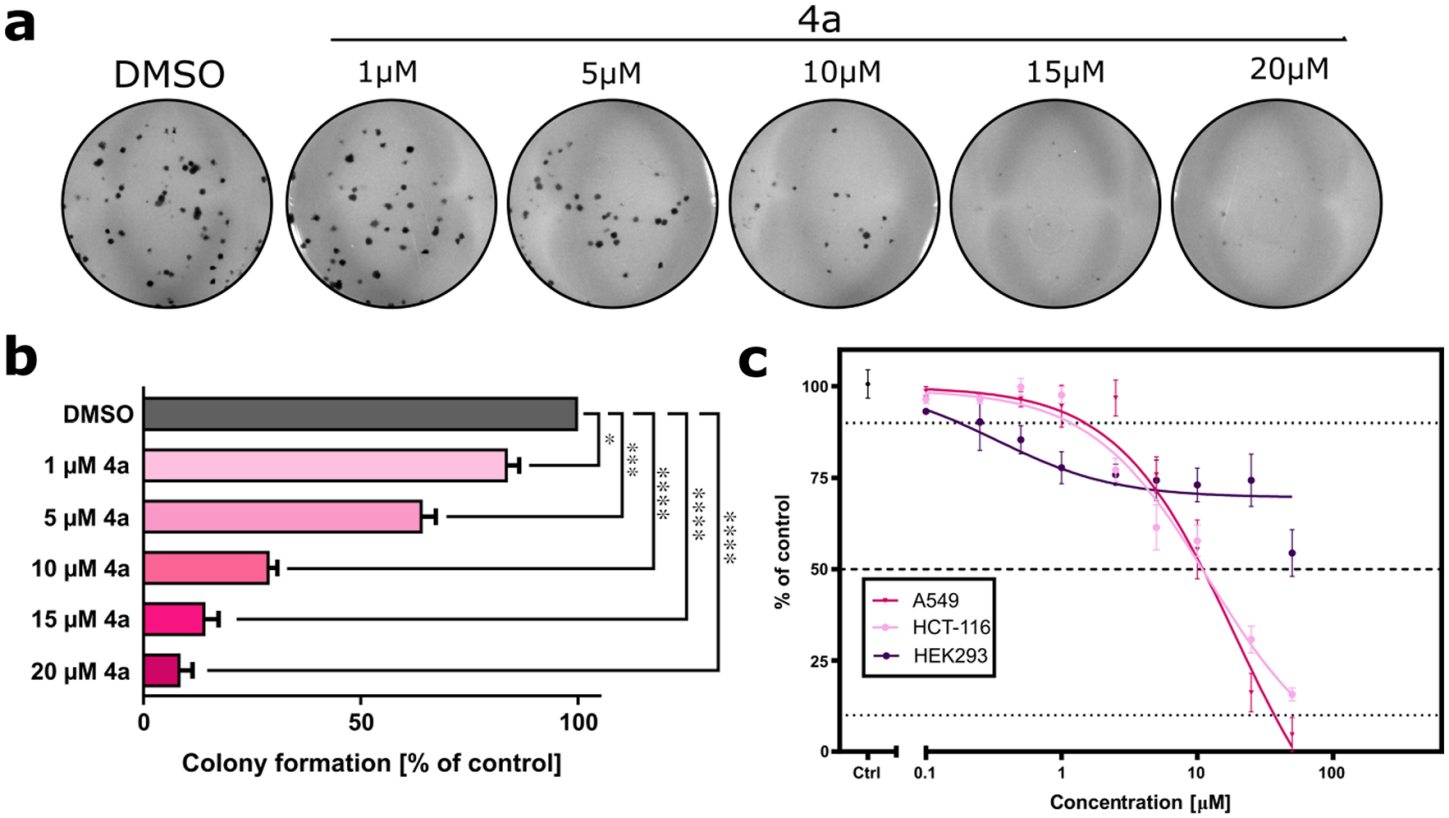
Effect of **4a** on colony formation and cell viability. (**a**) Representative photos of the colony formation assay after the treatment of A549 cells with increasing concentrations of **4a**. (**b**) Quantification of the colony formation assay. (**c**) Effect of **4a** on cell viability after incubation with compound for 72 h. Data represent the mean ± SEM of n = 3 independent experiments. *p < 0.01, **p < 0.001, ***p < 0.0001, and ****p < 0.00001.

All new tetrahydroquinoline derivatives were assessed for cytotoxicity on a broad panel of cancer and normal cell lines, using the MTT assay. The half-inhibitory growth inhibitory concentration (IC_50_) values of each compound were calculated after 72 h of treatment and presented in Table 1. The results of the biological evaluation showed that compounds **4b**, **4c**, **4d** and **4e** did not have any effect on the tested cell lines in the range of 0–50 µM, whereas compounds **5**, **4a**, and **6** significantly decreased the viability of colon cancer cells (HCT-116) with an IC_50_ value of approximately 13 µM. Additionally, **4a** and **6** (A549) had an IC_50_ value of 11.33 ± 0.67 and 40.18 ± 0.94 µM, respectively, and exhibited almost no suppressive effect on human normal HEK293 kidney cells in tested concentrations compared to the effect of compounds on A549 and HCT-116 cells (Fig. 3). In this assay, the IC_50_ value of 5-FU and ETP was recorded as a positive control. Based on the observations, **4a** was recognized as the most potent, and was therefore chosen for further analysis of the mechanisms underlying the antiproliferative action.

**Table 1:** Cytotoxic activity of tetrahydroquinoline derivatives against various cancer and normal cells presented as an IC_50_value referring to the concentration that inhibits 50% of cell growth.

Compound	Cell lines					
	HCT-116	A549	MCF-7	HepG2	Hela	HEK293
	IC_50_ (µM)					
**5**	13.10 ± 0.96	> 50	> 50	> 50	> 50	> 50
**4a**	12.18 ± 1.61****	11.33 ± 0.67****	> 50	> 50	> 50	49.01 ± 2.21
**6**	15.61 ± 1.29	40.18 ± 0.94	> 50	> 50	50	> 50
**4b**	> 50	> 50	> 50	> 50	> 50	> 50
**4c**	> 50	> 50	> 50	> 50	> 50	> 50
**4d**	> 50	> 50	> 50	> 50	> 50	> 50
**4e**	> 50	> 50	> 50	> 50	> 50	> 50
**5-FU**	5.78 ± 0.38****	8.12 ± 1.01***	8.41 ± 0.98**	13.12 ± 0.32*	11.09 ± 1.53*	16.82 ± 0.34
**ETP**	0.39 ± 0.01^ns^	0.54 ± 0.21^ns^	0.83 ± 0.15^ns^	5.01 ± 0.22^ns^	3.40 ± 0.51^ns^	1.91 ± 0.97

Data are expressed as the mean ± SD (µM) of n = 3 independent experiments. ****p < 0.00001 in comparison to IC_50_ for HEK293 (Student’s t-test).

Next, the effect of **4a** on the number of cell-forming clones was examined by the colony formation assay. As shown in Fig. 3, the number of observed colonies was found to be significantly decreased after treatment in comparison to the DMSO-treated control, and this effect was dose-dependent.

#### *Compound ****4a**** induces sub-G*_*1*_*-phase cell cycle arrest in A549 cells*

**Figure 4:**
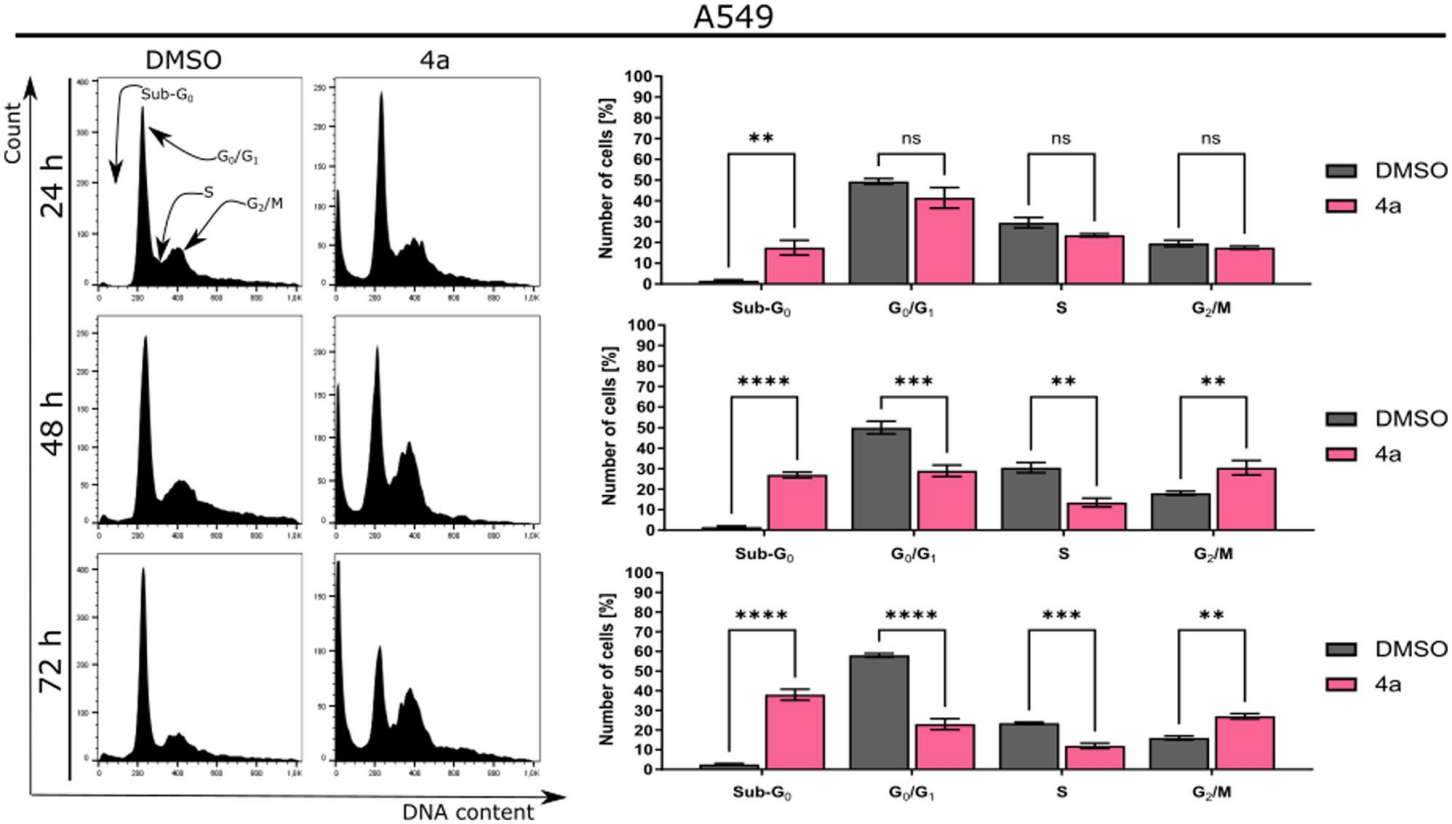
Analysis of cell cycle distribution after treatment of A549 cells with **4a**. Data represent the mean ± SEM of n = 3 independent experiments. *p < 0.01, **p < 0.001, ***p < 0.0001, and ****p < 0.00001.

The antiproliferative effect of **4a** was further assessed by examining the progression of the cell cycle (Fig. 4). The tested compound induced cell cycle arrest at the G_2_ phase in A549 cells. The fraction of cells in this phase increased significantly from 3.2% (DMSO-treated cells) to 15.5% and 33.8% (cells treated with the compound for 24 and 48 h, respectively). The observed changes corresponded with a time-dependent decrease in the fraction of cells at the G_0_/G_1_ phase and a concomitant increase in the sub-G_0_ phase.

#### Compound *4a* induces caspase-dependent apoptosis in A549 cells

**Figure 5:**
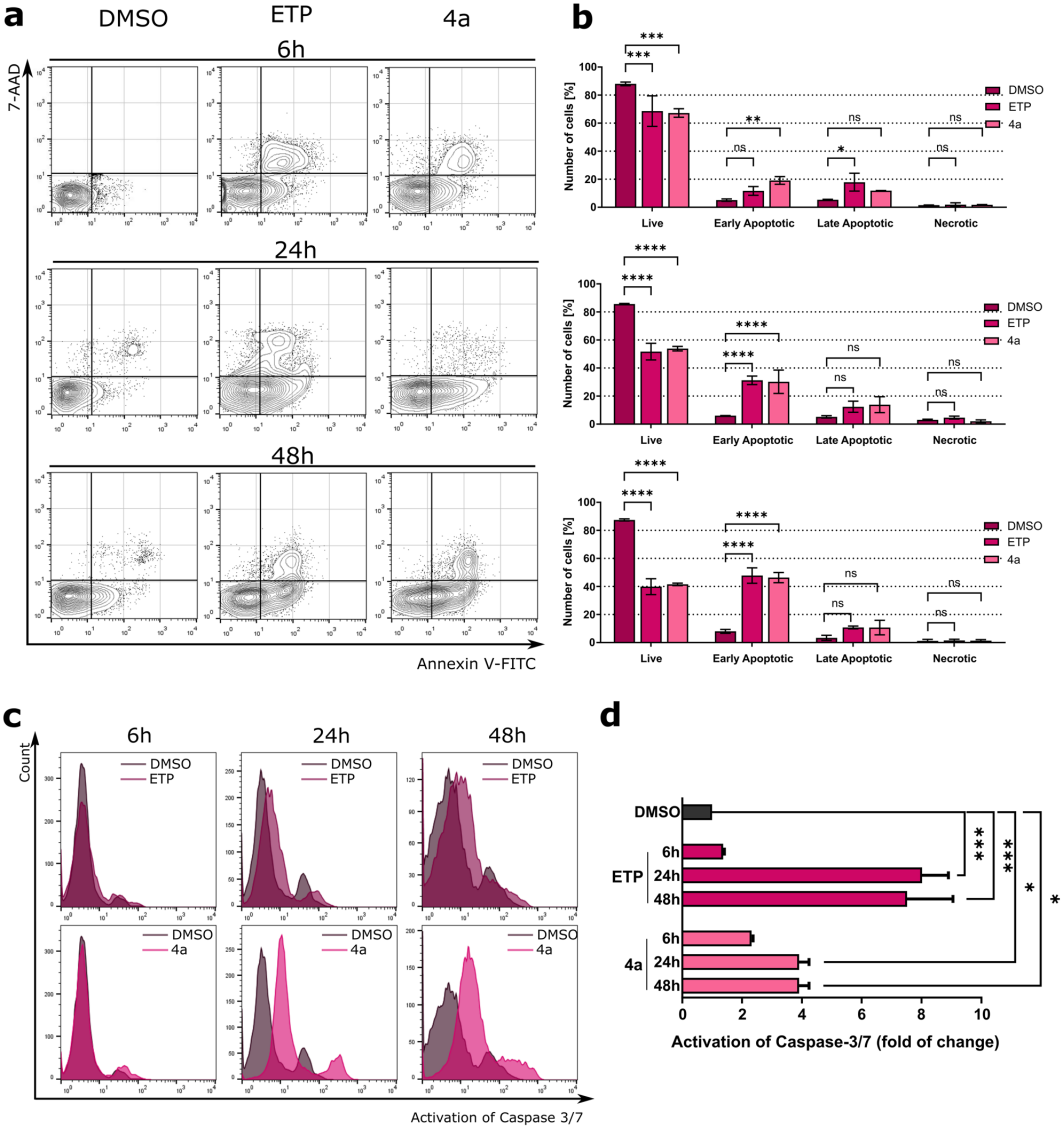
Detection of apoptosis by flow cytometry. (**a**) Representative dot plots after treatment of A549 cells with **4a**. (**b**) Quantitation of A549 cells after Annexin V-FITC/7-AAD staining. (**c**) Detection of caspase-3/7 activation in **4a**-treated A549 cells, presented as representative histograms. (**d**) Quantification of analysis from (**c**). Data represent the mean ± SEM of n = 3 independent experiments. *p < 0.01, **p < 0.001, ***p < 0.0001, and ****p < 0.00001.

To further explain the mechanism by which **4a** induced cell death in cancer cells, a flow cytometric analysis was performed by dual-staining the cells with 7-aminoactinomycin D (7-AAD) and Annexin V-fluorescein isothiocyanate (FITC). Annexin-V is a Ca^2+^-dependent protein with potent affinity to phosphatidylserine^24^. During early apoptosis, cells lose plasma membrane asymmetry through, for example, the externalization of phosphatidylserine. Once exposed to the outside of the cell surface, phosphatidylserine can be recognized by Annexin-V, and quantified^25^. On the other hand, 7-AAD is a dye that cannot penetrate the intact cell membrane, and allows differentiating between necrotic and late apoptotic cells from living and early apoptotic cells. As shown in Fig. 5, exposure of A549 cells to **4a** at its IC_50_ concentration for 6 h caused a significant decrease in the percentage of surviving cells (20.76 ± 1.24%, *p* = 0.0004), with a simultaneous increase in the fraction of early-apoptotic cells [7-AAD (–), Annexin V-FITC (+); 14.03 ± 1.74%, *p* = 0.0078] in comparison to DMSO-treated control cells (Fig. 5). Treatment with the tested compound for 24 h caused 3.9-fold augmentation in apoptosis [early: 7-AAD (–), Annexin V-FITC (+); late: 7-AAD (+), Annexin V-FITC (+)] in comparison to the vehicle. Further incubation of cells with **4a** for 48 h led to a significant increase in the percentage of apoptotic cells (*p* < 0.0001), while the level of the necrotic fraction was similar to that in control.

#### *Compound ****4a**** induces apoptosis *via* intrinsic and extrinsic pathways*

Apoptosis induction via both intrinsic and extrinsic pathways is mediated by caspase cascade events^26^. Therefore, in this study, the ability of **4a** to activate two effector caspases (caspase-3 and caspase-7) was evaluated by flow cytometry. As shown in Fig. 5, after 6 h of exposure to this compound, a slight increase (2.32 ± 0.05-fold) in the activity of caspase-3/7 was observed in A549-treated cells. After 24 and 48 h of incubation, remarkable activation of caspase-3/7 (3.9 ± 0.33-fold, *p* = 0.04) was observed in comparison to the vehicle.

**Figure 6:**
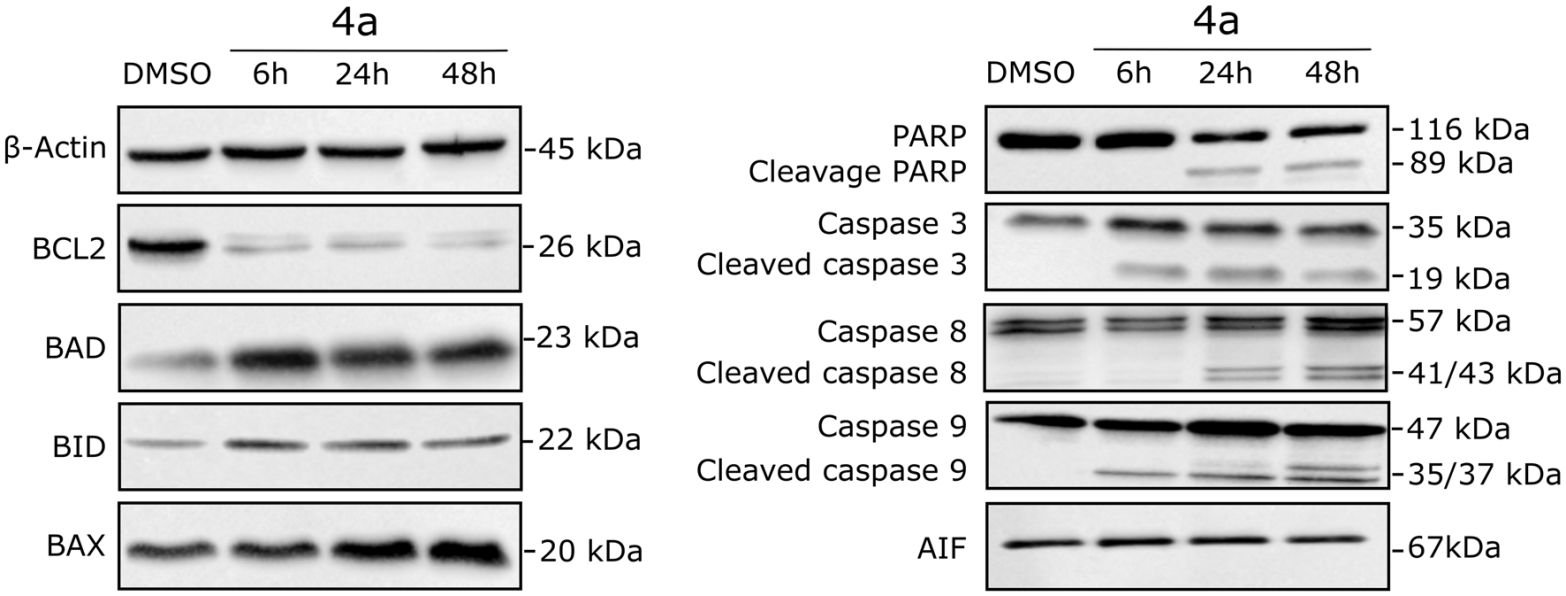
Western blotting analysis showing the effects of **4a** on the expression of apoptosis-related proteins in the A549 cell line. Full-length Western blots are presented in the supplementary information.

Furthermore, the expression of several apoptosis-related proteins was measured by Western blotting. As shown in Fig. 6, treatment of A549 cells with **4a** resulted in time-dependent modulation of expression of all investigated proteins. The expression of BCL-2 protein was significantly decreased, while the expression of BID was slightly activated and that of BAD and BAX protein was higher than that in the control group. Treatment with **4a** also led to the disappearance of the band corresponding to intact PARP-1 resulting in its proteolytic cleavage and the formation of an 85-kDa fragment. In addition, all the examined caspases (caspase-3, caspase-8, and caspase-9) were cleaved into their corresponding catalytically active forms. Expression of AIF remained unchanged in comparison to DMSO-treated control, which confirmed that treatment with **4a** induced caspase-dependent cell death^27^.

### Structure–activity relationship (SAR)

**Figure 7:**
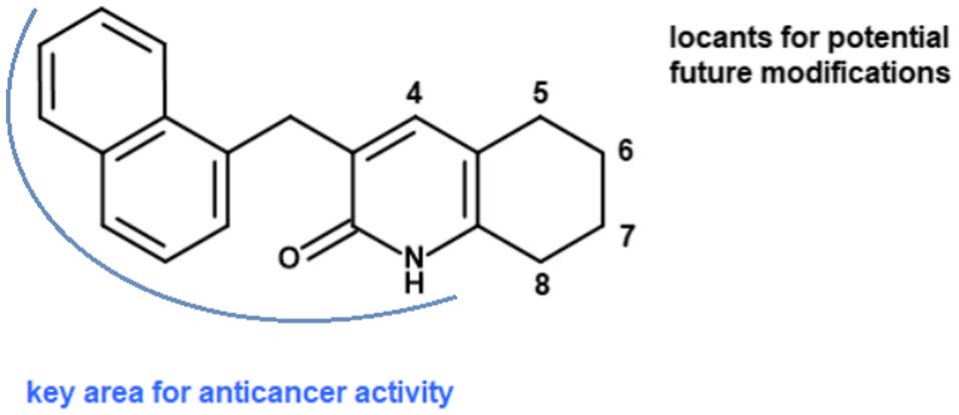
Conclusion of structure–activity relationship studies of the presented tetrahydroquinolinone derivatives.

During our work, we noticed that the IC_50_ values of presented compounds determined for HCT-116 and A549 strongly suggested that the (1-naphthyl)methyl moiety present at position 3 was crucial for the anticancer activity of the synthesized tetrahydroquinolinone derivatives (Fig. 7). Among the synthesized compounds, the best result was achieved for compound **4a** with the amide group. Modifications of compounds at position 2 with a chlorine atom or methoxy group increased their IC_50_ values. Therefore, alternatively, any other functionalization should be performed in the cyclohexyl ring or at position 4. The presented conclusions should be considered while designing subsequent derivatives from this subgroup.

## Discussion

From the perspective of the latest published data, it is worth noticing that new small-molecule compounds from the 2- and 4-quinolones group, which are often structurally based on antibacterial fluoroquinolones manifest an attractive activity in many cancer cell lines. It’s likely that compounds containing similar quinolone scaffold will exhibit activity towards the same cancer cell lines. For example derivatives with functionalysed quinolone nitrogen proposed by A.M.Y . Moustafa & S.B. Bakare and Zhou P. et al*.* were active toward hepatocellular cell line, whereas our molecule with free NH was active against non-small lung cancer cells. From the chemical point of view reservoir of possible functionalizations of different quinolone cores, seems to be very wide. The synthesis of compounds presented by A.M.Y. Moustafa & S.B. Bakare or Zhou P. et al*.* is easy to perform and gives a rich spectrum of interesting derivatives. For this reason, it’s encouraging and justified to use brief synthetic solutions more and more often. A very important aspect pointed out by Zhou P. et al*.* was related to new 7-chloro-4(1H)-quinolone derivatives. Due to the poor solubility of the initial compound in water which was probably responsible for its weak antiproliferative activity scientists paid attention to the ClogP value of each compound. The results show that the ClogP values of whole molecules could be a helpful factor in providing the antiproliferative properties of compounds and we will consider that in the future. Interesting and nonobvious functionalization of 2-quinolinone was also proposed by A.M.Y. Moustafa & S.B. Bakare and A. H. Abu Almaaty et al*.* Both research groups decided to bromine 4-methylquinolin-2(1H)-one derivative which isn’t a modification of the first choice. The introduction of halides into chemical structure increases the hydrophobicity of compounds which is usually an undesirable effect. Fortunately, researchers found an effective compromise between the potential reduction of solubility and still high biological activity of design compounds which led to obtaining IC_50_ values comparable to Doxorubicin and Staurosporine. That encourages us to further development of presented compounds by choosing less standard solutions. During our synthesis, the most problematic stage was amonolysis of benzyl-type derivatives of ethyl benzoylacetate which required a long reaction time and its yields were often unsatisfactory. That difficulty occurred most probably because of sterical hindrance and enoliztion of used compounds. Due to the fact that we’re interested in this type of condensations, it is possible that in the near future we will propose some synthetic paths which will solve this problem. Over the last several years, there is an increasing need for anticancer drugs that can perturb the proliferation of tumor cells by inhibiting various stages of the cell cycle^28^. In the present study, we examined the effect of compound **4a** on the progression of the cell cycle and observed that it arrested the cell cycle at the G_2_/M phase in A549 cells, leading to cell apoptotic cell death.. Antimitotic properties of quinoline-based compounds were also observed by others: e.g. Metwally et al*.* showed that pyrimido[4,5-c]quinoline-1(2H)-one ring skeleton exhibited high cytotoxicity towards fibrosarcoma HT-1080 cells inducing G_2_/M arrest and apoptosis, whereas a Yang`s group presented quinoline derivative MPT0B392 as a strong antimitotic agent against acute leukemia^29,30^. Many studies reported that the antimitotic activity of quinolone results from their interaction with tubulin at the colchicine-binding site, disrupting microtubule organization; however, in the case of the **4a,** the exact mechanism of blocking the G_2_/M cell cycle remains unresolved and will be subject of the future studies^31–34^. Caspase-3 and caspase-7 are members of the cysteine-aspartic acid protease family, which play an important role in executing apoptotic death^35^. Once activated, both these enzymes cleave multiple key structural and repair proteins. Compound **4a** stimulated the activation of both caspase-3 and caspase-7, which could have triggered the antiproliferative effect on A549 cells. Caspase activation in response to anticancer chemotherapy can occur via two major pathways: intrinsic pathway mediated by mitochondria, or extrinsic pathway mediated by receptors. The mitochondria-mediated pathway is initiated by the activation of proapoptotic BCL-2 effector molecules, such as BAX and BAK, which leads to the permeabilization of mitochondrial outer membrane and subsequently the release of cytochrome c from the mitochondrial intermembrane space to the cytosol. Once released, it binds to its partner, Apaf-1 protein, forming an apoptosome complex, which induces the activation of pro-caspase-9^36^. The extrinsic pathway is activated by the stimulation of death receptors (e.g. Fas (CD95/APO-1)), TNF receptors (e.g. TNF/R1/p55/CD120), or TNF-related apoptosis-inducing ligand^37^. Ligation of TNF-receptor family proteins results in the assembly of DISC, and the subsequent recruitment of caspase-8 which occurs through the interaction between the death effector domain of caspase 8 and the Fas-associated death domain. Then, caspase 8 is oligomerized and activated by self-cleavage, after which it initiates downstream apoptotic events, including the activation of effector caspases such as caspase-3^38^.

The results of Western blotting indicated that exposure of A549 cells to **4a** diminished the expression of the antiapoptotic protein BCL-2, while enhancing the expression of the proapoptotic protein BAX, in comparison to the control group. Moreover, the BAX:BCL-2 ratio was significantly increased, which suggests the permeabilization of the mitochondrial outer membrane^39^. The level of the proapoptotic molecule BAD was also elevated upon treatment with **4a**. Huang et al*.* reported that the loss of BAD expression may indicate a poor prognosis in patients with nonsmall cell lung cancer, due to increased resistance to the treatment^40^. Therefore, high BAD expression may be considered to suppress tumorigenesis. In this study, together, with the modulating members of the BCL-2 family, treatment with **4a** led to the cleavage of procaspase-9, promoting mitochondria-mediated apoptosis. However, the levels of cleaved caspase-8, which is the initiator caspase of the extrinsic apoptotic pathway, were also increased in the treated cells in comparison to the control cells^41^. Additionally, exposure to **4a** induced proteolytic cleavage of PARP-1, which catalyzes the transfer of ADP-ribose polymers onto itself and the proteins activated in response to double-strand DNA breaks. It should be mentioned that cleavage of PARP-1, which is initiated by caspase-3 and caspase-7, is known as a hallmark of apoptosis^42^.

Activation of both intrinsic and extrinsic apoptotic pathways by quinolinone was also reported by Janecka`s group, which showed that 2-ethyl-3-methyliden-1-tosyl-2,3-dihydroquinolin-4-(1H)-one exhibited cytotoxic activity by down-regulation MAPK pathway in myeloid leukemia HL-60 cell line^43^. On the other hand, Beretta et al*.* revealed that cytotoxic properties of quinoline–pyrrolidine gamma-lactam alkaloid towards prostate cancer cells result from activation of transmembrane receptor-mediated interactions^44^; whereas Chiu et al*.* showed that quinoline derivative BPIQ induced mitochondrial apoptosis in lung cancer models^45^. Potent anticancer activities of quinolones have been found against various cancer cell lines and targets, including topoisomerase, protein tyrosine kinases, histone deacetylase, and many others^46^. Interestingly, inhibition of all mentioned targets may trigger G_2_/M cell cycle arrest and apoptotic cell death, similar to **4a**. In conclusion, the newly synthesized tetrahydroquinolinone derivatives containing (1-naphthyl)methyl moiety at position 3 reduced the activity of non-small cell lung cancer cells in a time-dependent manner. Among them, compound **4a** inhibited colony formation and proliferation of A549 cells. Further analysis of this compound revealed that it induced apoptosis through both intrinsic and extrinsic apoptotic pathways. These findings may be valuable for the future development of anticancer agents based on tetrahydroquinolinone derivatives.

## Methods

### Cell cultivation

Non-small cell lung cancer cells A549 (CCL-185), breast cancer cells MCF-7 (HB-8065), hepatocarcinoma cells HepG2 (HB-8065), normal kidney cells HEK293 (CRL-1573), and osteosarcoma cells U-2 OS (HTB-96) were obtained from the American Type Culture Collection. HCT-116 cell line was kindly provided by prof. Bert Vogelstein (Johns Hopkins University, Howard Hughes Medical Institute, USA). The obtained A549 and MCF-7 cells were cultured in RPMI-1640 medium, HepG2 cells in Minimum Essential Medium Eagle medium, HEK-293 cells in Dulbecco’s Modified Eagle’s medium, and U-2 OS and HCT-116 cells in McCoy’s **4a** medium. All culture media were supplemented with 10% fetal bovine serum, 2 mM L-glutamine, and antibiotics (penicillin 62.6 µg/ml and streptomycin 40 µg/ml; Sigma-Aldrich). Both cancer and normal cells were cultured at 37 °C under a humidified atmosphere with 5% or 10% CO_2_ and routinely screened for *Mycoplasma* contamination. All materials and reagents were obtained from Corning unless stated otherwise.

### Cell viability assay

The cytotoxic effect of the synthesized compounds was assessed by the MTT (4,5-dimethylthiazol-2-yl-2,5-diphenyltetrazoliumbromide; Sigma-Aldrich) assay. Briefly, the cells were seeded into 96-well transparent flat-bottom plates and allowed to attach overnight. On the next day, the cells were treated with compounds in the range of 0–50 µM for 72 h. Dimethyl sulfoxide (DMSO; Merck) and 5-fluorouracil (5-FU; Sigma-Aldrich) were used as a reference. After exposure to the compounds, 20 μl of the MTT solution at a concentration of 4 mg/ml was added to each well, and the plates were incubated again for 2–3 h at 37 °C. Next, the media in the wells was replaced with 100 μl DMSO to solubilize the formazan crystals. The absorbance was measured at 540 nm using an ASYS UVM340 microplate reader (Biochrom Ltd.). The IC_50_ value of the compounds was calculated using GraphPad Prism 9 software, based on the curves plotted with survival as a function of dose, averaged from three independent experiments.

### Colony formation assay

HCT-116 and A549 cells were seeded into six-well plates at a density of 500 cells per well and allowed to attach overnight. On the next day, the cells were pretreated with compound **4a** at different concentrations or with 1% v/v DMSO for 24 h. After incubation, the cells were washed and cultured for 9 days. Next, the cells were fixed with methanol for 20 min and stained with 0.5% crystal violet (Sigma-Aldrich). Finally, the plates were dried overnight and imaged using a UVITEC Cambridge imaging system. Quantification was performed with ImageJ software. The surviving fraction was calculated as previously described^47^. Each experiment was repeated three times.

### Cell cycle progression

A549 and HCT-116 cells were incubated with the tested compounds at their IC_50_ concentrations for 24, 48, and 72 h. DMSO (1% v/v) was used as a reference (Sigma-Aldrich). After incubation, the cells were collected in trypsin, fixed with 75% ice-cold ethanol, and stored overnight at − 20 °C. Then, the cells were stained with 20 μg/ml propidium iodide (Sigma-Aldrich) and 50 μg/ml RNaseA (Thermo Fisher Scientific) in phosphate-buffered saline (PBS) for 30 min at room temperature (RT). The content of DNA in cells was determined by flow cytometry (Guava EasyCyte 8 cell sorter; Merck Millipore) using FlowJo software v10. Each experiment was repeated three times.

### Apoptosis and caspase-3/7 assay

Cells were seeded in a 5-cm^2^ dish at a density of 15 × 10^5^ cells per dish and allowed to attach overnight at 37 °C. Then, compounds at IC_50_ concentrations were added to the medium. After 6, 24, and 48 h, the cells were harvested and washed with PBS. Subsequently, the cells were resuspended in the binding buffer and stained with Annexin V Alexa Fluor™ 488 conjugate (#A13201; Thermo Fisher Scientific) for the apoptosis assay and with CellEvent™ Caspase-3/7 Green Flow Cytometry Assay Kit (#C10427; Thermo Fisher Scientific) for the activation of caspase-3/7, as per the manufacturer’s instructions. After incubation, the samples were immediately analyzed using a Guava EasyCyte 8 cell sorter (Merck Millipore) and FlowJo software v10. Each experiment was repeated three times.

### Western blotting

The expression of apoptosis-related proteins was determined by Western blotting. Briefly, A549 cells were treated with compound **4a** at its IC_50_ concentration for 6, 24, and 48 h. The cells treated with 1% v/v DMSO served as the negative control. The total protein content was extracted from the cells using the NP-40 cell lysis buffer (10 mM Tris–HCl pH 7.4, 10 mM NaCl, 3 mM MgCl_2_, 0.5% Nonidet P-40, cOmplete Mini EDTA-free Protease Inhibitor Cocktail). Then, 30 µg of protein extract was separated by 10% sodium dodecyl sulfate–polyacrylamide gel electrophoresis, and the separated proteins were transferred to microporous polyvinylidene difluoride membranes (Bio-Rad). The membranes were blocked with 5% bovine serum albumin in tris-buffered saline-Tween 20 (TBST) buffer (0.2 M Tris-base, 0.137 M NaCl, 0.1% Tween 20) for 1 h at RT, and incubated overnight with primary antibodies at 4 °C. The blots were washed three times with TBST and incubated with appropriate peroxidase-conjugated secondary antibodies for 1 h at RT. Then, the blots were washed in TBST for 30 min and developed using an enhanced chemiluminescence detection reagent kit (Thermo Fisher Scientific) and a ChemiDoc XRS + Imaging System (Bio-Rad). All materials and reagents were obtained from Sigma-Aldrich unless stated otherwise. The antibodies used in Western blotting are listed in Table S1.

### Statistical analyses

Statistical analyses were performed using GraphPad Prism 9 software. Data were obtained from at least three independent experiments and are presented as mean ± SEM. Statistical significance was calculated in comparison to the DMSO-treated control (1% v/v) using one-way ANOVA (post hoc Dunnet’s test) unless stated otherwise.

## Supplementary Information

Supplementary Information.

## Data Availability

The datasets presented in the current study are available from the corresponding author on reasonable request.
