# Supplementary material for: Tetrahydroquinolinone derivatives exert antiproliferative effect on lung cancer cells through apoptosis induction
Source: Sci Rep. 2022 Nov 9;12:19076. doi: 10.1038/s41598-022-23640-9 (PMC9646836; doi:10.1038/s41598-022-23640-9)
Supplement: Supplementary file 1 — Supplementary Information. [file 41598_2022_23640_MOESM1_ESM.docx]

**SUPPLEMENTARY INFORMATION**

**Tetrahydroquinolinone derivatives exert antiproliferative effect on lung cancer cells through apoptosis induction**

**Małgorzata Ryczkowska^1^, Natalia Maciejewska^2^, Mateusz Olszewski^2^, Milena Witkowska^1^, and Sławomir Makowiec^1^***

^1^ Department of Organic Chemistry, Faculty of Chemistry, Gdansk University of Technology, Narutowicza 11/12, 80-233, Gdansk, Poland.

^2^ Department of Pharmaceutical Technology and Biochemistry, Faculty of Chemistry, Gdansk University of Technology, Narutowicza 11/12, 80-233, Gdansk, Poland.*mak@pg.edu.pl

**EXPERIMENTAL DATA**

**A. General procedure for benzyl-type derivatives of ethyl benzoylacetate**

A solution of ethyl benzoylacetate (0.63 mmol) and K_2_CO_3_ (0.95 mmol) in 3 ml of DMF was placed in round-bottomed flask with stir bar. Corresponding benzyl halide (0.82 mmol) was added in one portion. The reaction mixture was heated to 60^o^C and left for 4h. DMF was evaporated. The residue was dissolved with ethyl acetate and washed with water and brine. Organic layer was dried with anhydrous MgSO_4_. Crude products were isolated by flash column chromatography (ethyl acetate : hexane 1:40 + 2% toluene). Yields and characteristic of title compounds are collected below.

**1. ethyl 2-(1-naphthylmethyl)-3-oxo-3-phenyl-propanoate (2a)**

Colorless oil, yield: 67%,

^1^H NMR (CDCl_3_, 400 MHz): δ= 8.07 (d, *J* = 8.4 Hz, 1 H), 7.90 – 7.85 (m, 3 H), 7.71 (d, *J* = 8 Hz, 1 H), 7.57 – 7.48 (m, 3 H), 7.41 – 7.32 (m, 4 H), 4.82 (t, *J* = 7.2 Hz , 1 H), 4.14 – 4.05 (m, 2 H), 3.84 (d, *J* = 7.2 Hz, 2 H), 1.09 (t, *J* = 7.1 Hz, 3 H)

^13^C NMR (CDCl_3_, 100 MHz): δ= 194.75, 169.60, 136.40, 134.35, 134.03, 133.60, 131.77, 129.13, 128.73, 128.69, 127.63, 127.47, 126.38, 125.72, 125.55, 123.36, 61.69, 55.02, 31.82, 14.02

HRMS (ESI+): *m*/*z*

[M + H]+ calcd for C_22_H_21_O_3_: 333.1491; found: 333.1498

**
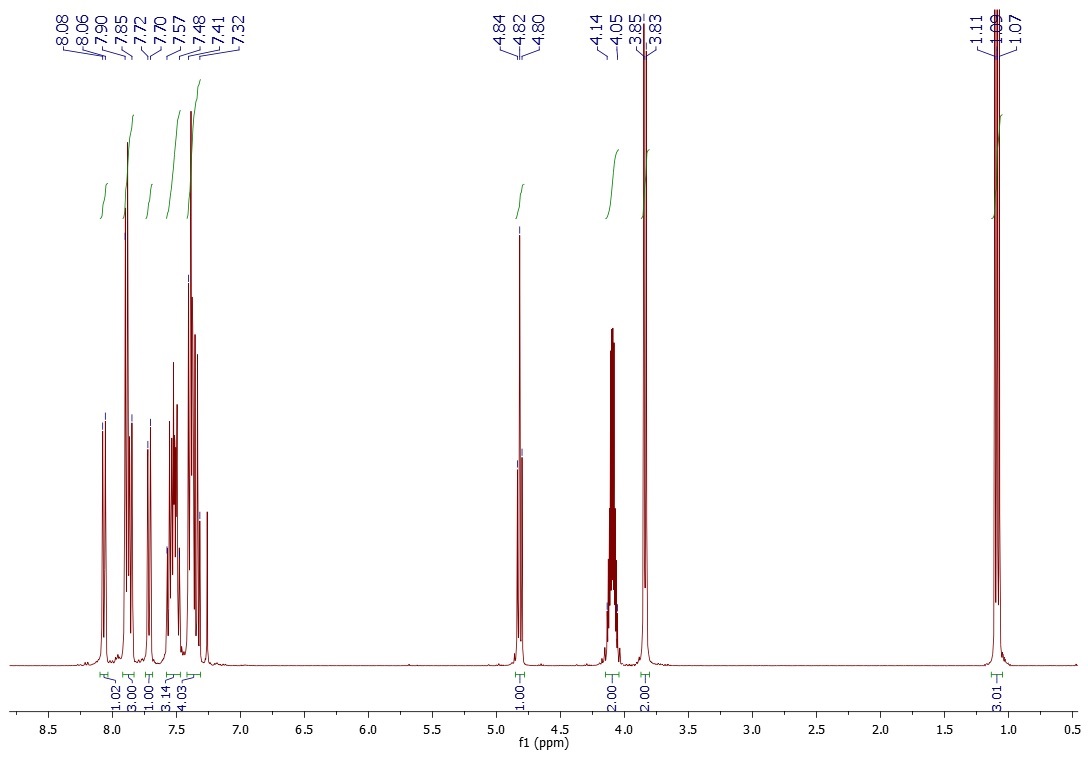
Figure S1. ^1^H NMR of ethyl 2-(1-naphthylmethyl)-3-oxo-3-phenyl-propanoate**

**
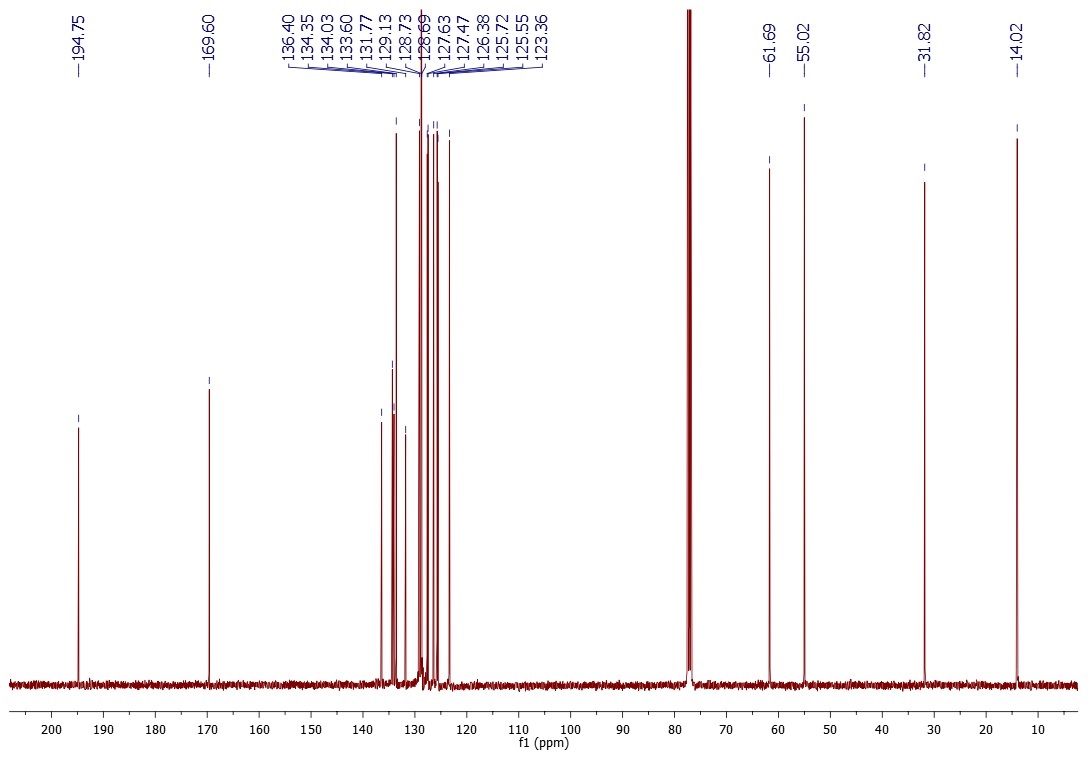
**

**Figure S2. ^13^C NMR of ethyl 2-(1-naphthylmethyl)-3-oxo-3-phenyl-propanoate**

**2. ethyl 2-(2-naphthylmethyl)-3-oxo-3-phenyl-propanoate (2b)**

Colorless oil, yield: 75%,

Mixture of tautomers 7:3

^1^H NMR (CDCl_3_, 400 MHz): δ= 7.99 – 7.97 (m, 1.86 H), 7.87 – 7.68 (m, 4 H), 7.62 – 7.36 (m, 6 H), 7.23 – 7.20 (m, 0.14 H), 4.73 (t, *J* = 7.3 Hz, 0.78 H), 4.30 – 4.19 (m, 0.36 H), 4.16 – 4.04 (m, 1.63 H), 3.99 (s, OH), 3.67 (d, *J* =14.1 Hz, 0.14 H), 3.58 (d, *J* = 14.1 Hz, 0.14 H) 3.69 – 3.42 (dd, *J^3^* = 7.3 Hz, *J^2^* = 2 Hz, 1.63 H), 1.26 (t, *J* = 7.1 Hz , 0.87 H), 1.10 (t, *J* = 7.1 Hz, 2.13 H)

^13^C NMR (CDCl_3_, 100 MHz): δ= 194.54, 169.40, 136.30, 136.09, 133.69, 133.61, 132.41, 128.84, 128.82, 128.33, 127.72, 127.64, 127.37, 126.17, 125.68, 61.70, 56.31, 35.02, 14.08

HRMS (ESI+): *m*/*z*

[M + H]+ calcd for C_22_H_21_O_3_: 333.1491; found: 333.1492

**
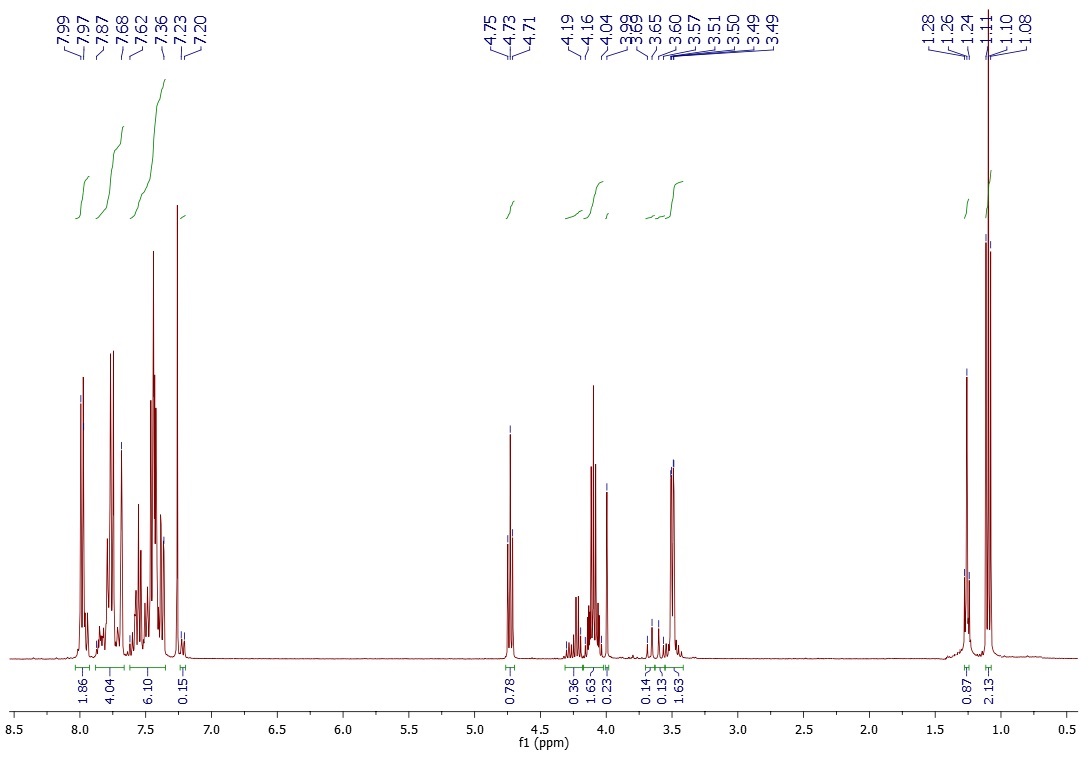
**

**Figure S3. ^1^H NMR of ethyl 2-(2-naphthylmethyl)-3-oxo-3-phenyl-propanoate**

**
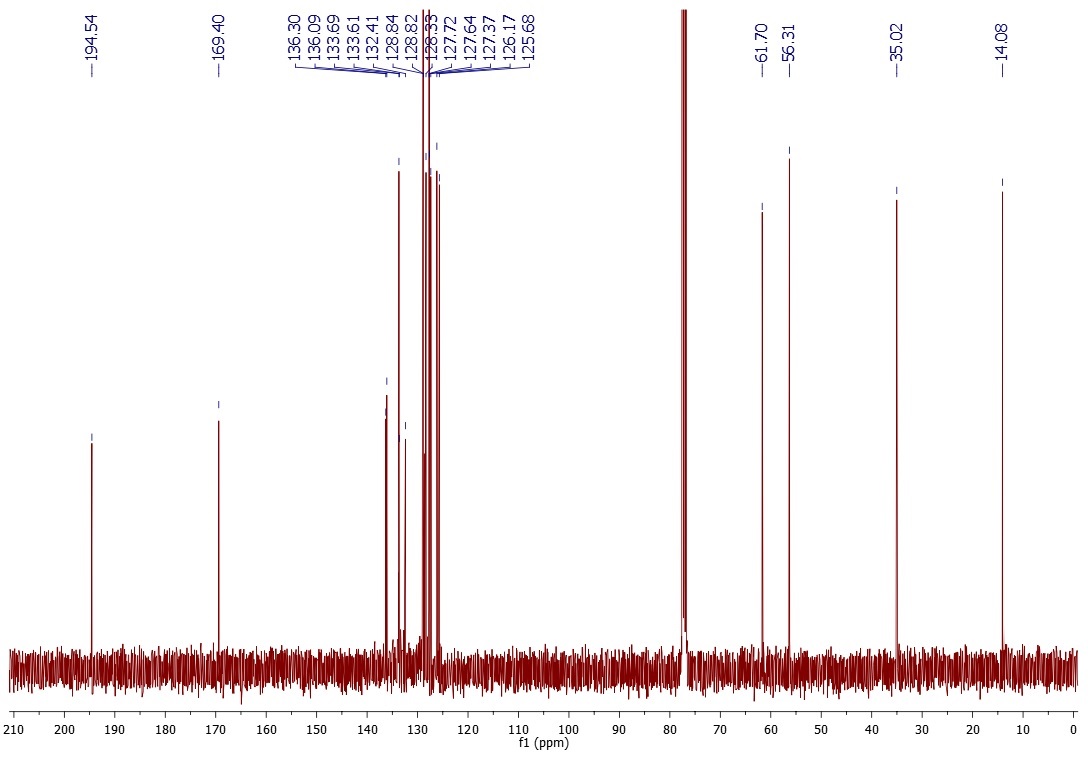
**

**Figure S4. ^13^C NMR of ethyl 2-(2-naphthylmethyl)-3-oxo-3-phenyl-propanoate**

**3. ethyl 3-oxo-3-phenyl-2-[(4-phenylphenyl)methyl]propanoate (2c)**

Colorless oil, yield: 85%,

^1^H NMR (CDCl_3_, 400 MHz): δ= 8.00 – 7.98 (m, 2 H), 7.59 – 7.54 (m, 3 H), 7.51 – 7.40 (m, 6 H), 7.34 – 7.30 (m, 3 H), 4.66 (t, *J* = 7.3, 1 H), 4.18 – 4.07 (m, 2 H), 3.38 (dd, *J^3^* = 7.3 Hz, *J^2^* = 1.7 Hz, 2 H), 2 H), 1.13 ( t, *J* = 7.1 Hz, 3 H)

^13^C NMR (CDCl_3_, 100 MHz): δ= 194.57, 169.41, 140.95, 139.68, 137.67, 136.31, 133.70, 129.50, 128.87, 128.85, 128.85, 127.37, 127.31, 127.11, 61.70, 56.28, 34.52, 14.09

HRMS (ESI+): *m*/*z*

[M + H]+ calcd for C_24_H_23_O_3_: 359.1647; found: 359.1651.


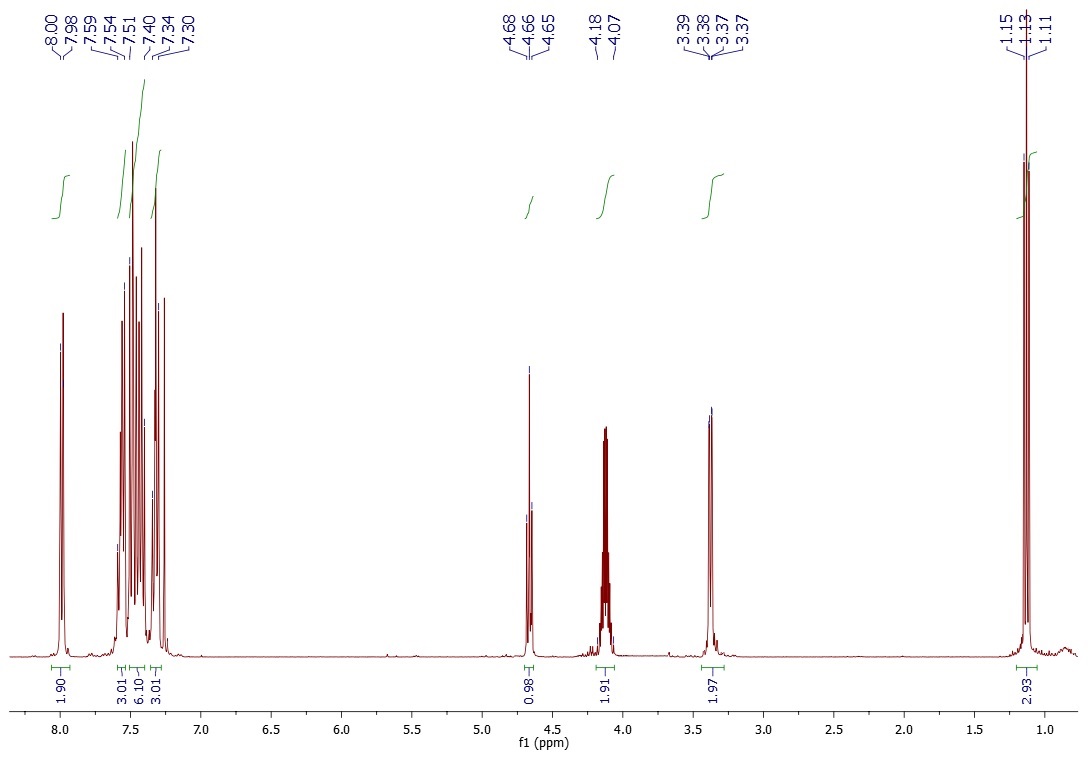


**Figure S5. ^1^H NMR of ethyl 3-oxo-3-phenyl-2-[(4-phenylphenyl)methyl]propanoate**

**
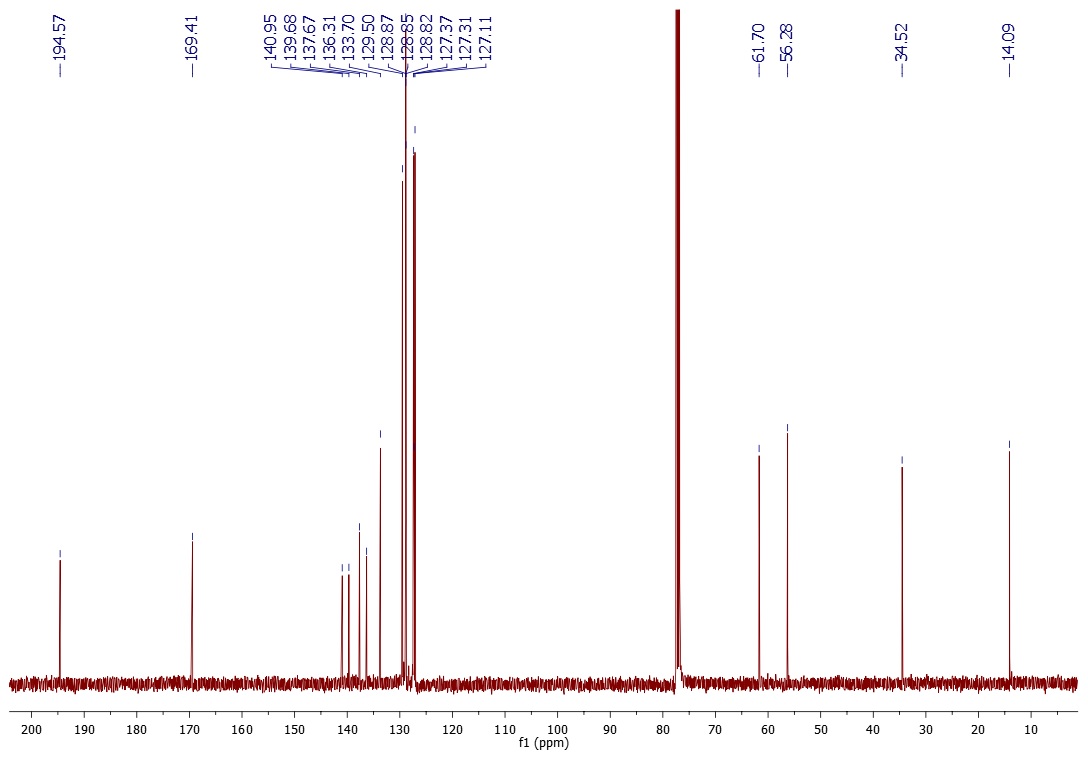
**

**Figure S6. ^13^C NMR of ethyl 3-oxo-3-phenyl-2-[(4-phenylphenyl)methyl]propanoate**

**4. ethyl 3-oxo-3-phenyl-2-[(3-phenylphenyl)methyl]propanoate (2d)**

Bright yellow oil, yield: 91%,

^1^H NMR (CDCl_3_, 400 MHz): δ= 7.99 – 7.96 (m, 2 H), 7.58 – 7.52 (m, 3 H), 7.47 – 7.41 (m, 6 H), 7.36 – 7.31 (m, 2 H), 7.23 – 7.21 (d, *J* = 7.6 Hz, 1 H), 4.68 (t, *J* = 7.3 Hz, 1 H), 4.15 – 4.07 (m, 2 H), 3.40 (dd, *J^2^* = 2.2 Hz, *J^3^* = 7.3 Hz, 2 H), 1.11 (t, *J* = 7.1 Hz, 3 H)

^13^C NMR (CDCl_3_, 100 MHz): δ= 194.61, 169.41, 141.59, 141.15, 139.06, 136.35, 133.68, 129.08, 128.84, 128.82, 128.01, 127.96, 127.43, 127.27, 125.65, 61.69, 56.26, 34.99, 14.08

HRMS (ESI+): *m*/*z*

[M + H]+ calcd for C_24_H_23_O_3_: 359.1647; found: 359.1646.

**
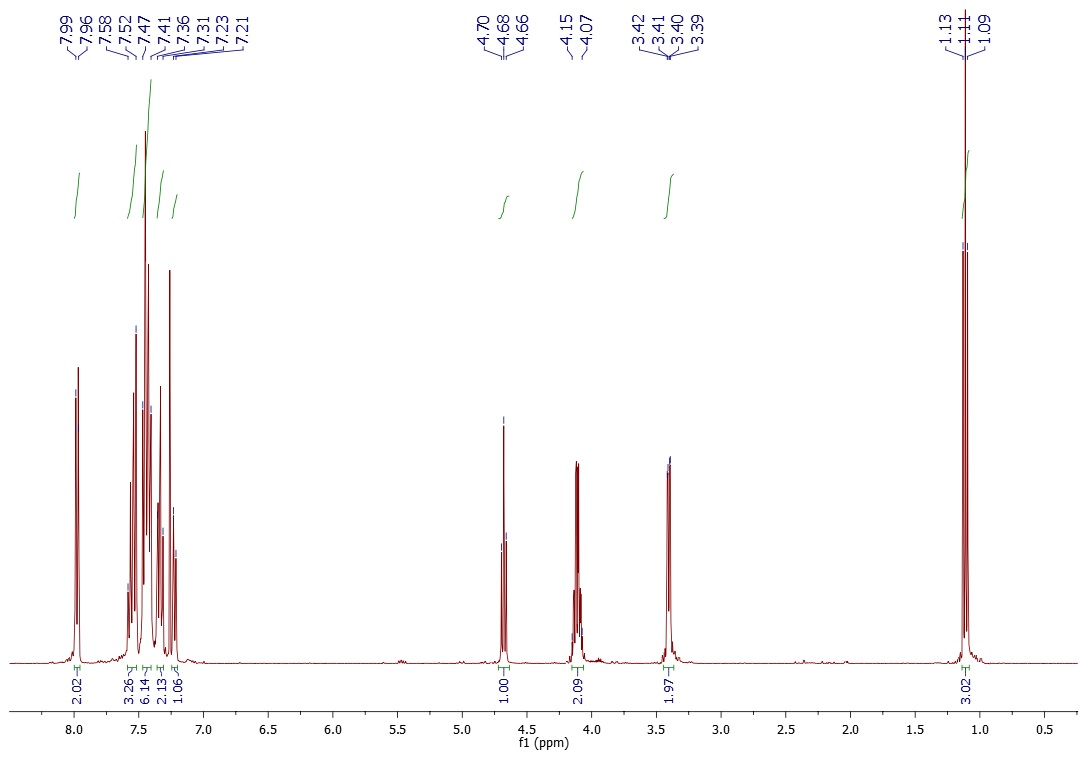
**

**Figure S7. ^1^H NMR of ethyl 3-oxo-3-phenyl-2-[(3-phenylphenyl)methyl]propanoate**

**
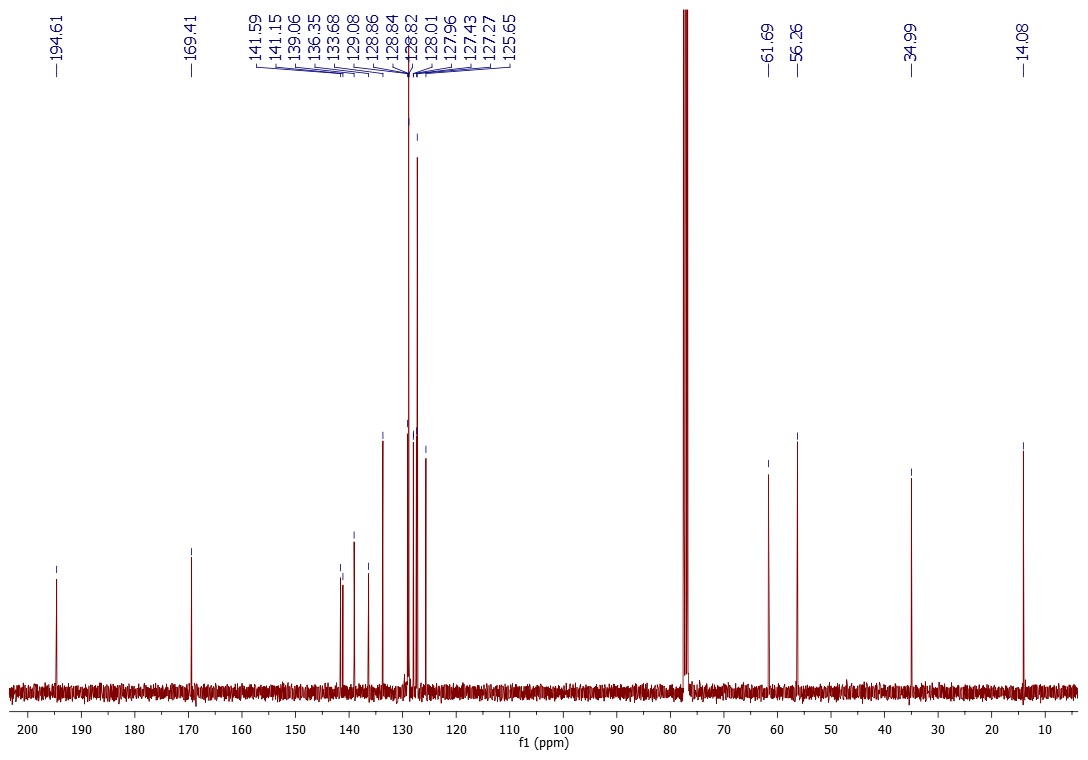
**

**Figure S8. ^13^C NMR of ethyl 3-oxo-3-phenyl-2-[(3-phenylphenyl)methyl]propanoate**

**7. ethyl 3-oxo-3-phenyl-2-[[4-(trifluoromethyl)phenyl]methyl]propanoate (2e)**

Colorless oil, yield: 65%,

Mixture of tautomers 1:9

^1^H NMR (CDCl_3_, 400 MHz): δ= 7.97 – 7.95 (m, 2 H), 7.60 – 7.56 (m, 1 H), 7.53 – 7. 44 (m, 4 H), 7.36 (d, *J* = 8.0 Hz, 2 H), 4.62 (t, *J* = 7.4 Hz, 1 H), 4.14 – 4.06 (m, 2 H), 3.38 (d, *J* =7.4 Hz, 2 H), 1.11 (t, *J* = 7.1 Hz, 3 H)

^13^C NMR (CDCl_3_, 100 MHz): δ= 194.00, 169.06, 142.69 (q, *J^5^* = 1,1Hz), 136.07, 133.88, 129.47, 129.16 (q, *J^2^* = 23.9 Hz), 128.91,128.78, 128.63, 125.57 (q, *J^3^*= 3.7 Hz), 124.27 (q, *J^1^* = 279, 4 Hz), 61.85, 55.84, 34.51, 14.02

HRMS (ESI+): *m*/*z*

[M + H]+ calcd for C_19_H_18_F_3_O_3_: 351.1208; found: 351.1204.


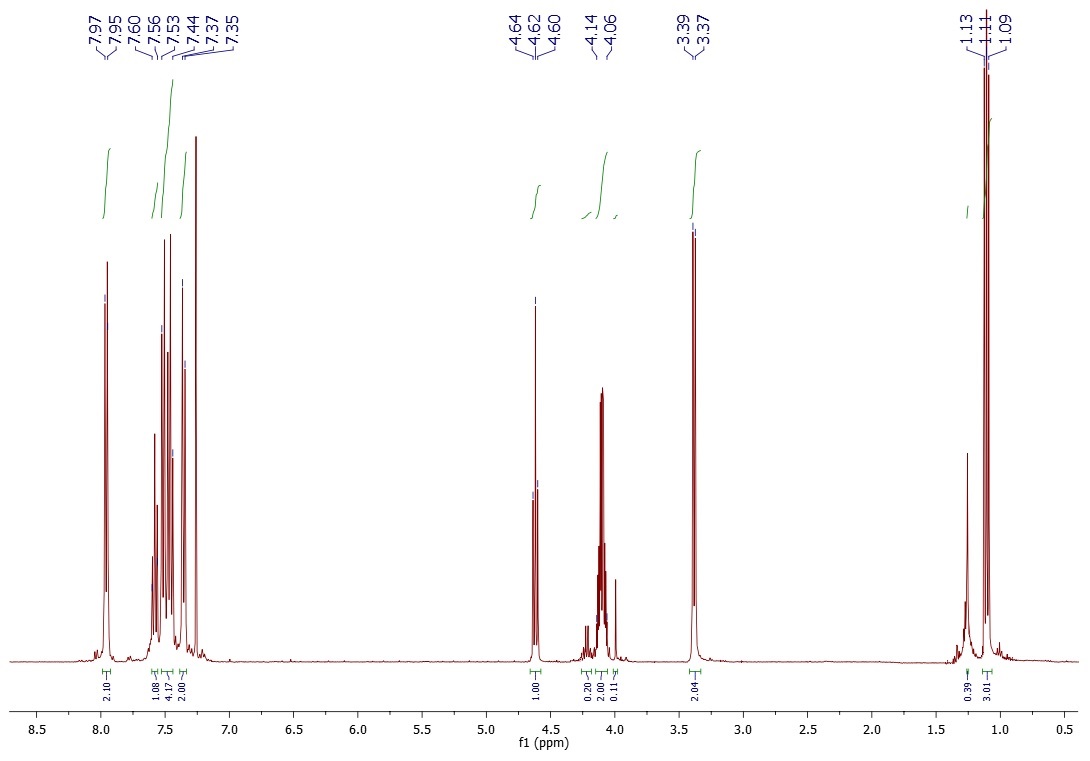


**Figure S9. ^1^H NMR of** **ethyl 3-oxo-3-phenyl-2-[[4-(trifluoromethyl)phenyl]methyl]propanoate**

**
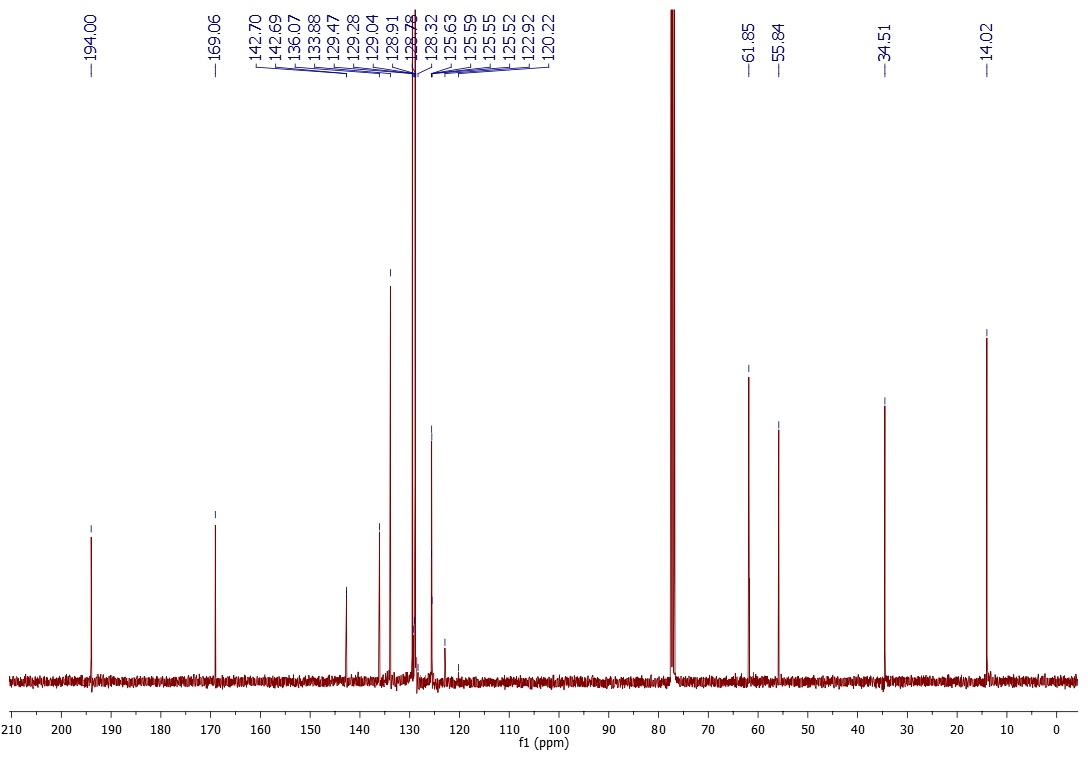
Figure S10. ^13^C NMR of** **ethyl 3-oxo-3-phenyl-2-[[4-(trifluoromethyl)phenyl]methyl]propanoate**

**B. General procedure for aromatic derivatives of 3-oxo-3-phenyl-propanamide [1]**

Benzyl-type derivatives of ethyl benzoylacetate **2a-e** (0.63 mmol) and 50 ml of 24% ammonia water were placed in round-bottomed flask with stir bar. The reaction temperature was raised to 45^o^C for 16h to 3 days depending on corresponding substrate and monitored by thin layer chromatography. After cooling to RT solvent was evaporated. Title products were purified by flash column chromatography (ethyl acetate : hexane 1:1). Yields and characteristic of title compounds are collected below.

**1. 2-(1-naphthylmethyl)-3-oxo-3-phenyl-propanamide (3a)**

White amorphous powder, yield: 33%,

^1^H NMR (CDCl_3_, 400 MHz): δ= 8.12 (d, *J* = 8.1 Hz, 1 H), 7.82 (d, *J* = 8.0 Hz, 1 H), 7.73 (d, *J* = 7.4 Hz, 1 H), 7.57 (t, *J* = 7.2 Hz, 1 H), 7.51 – 7.45 (m, 2 H), 7.31 – 7.24 (m, 4 H), 6.47 (s, NH), 5.62 (s, NH), 4.81 (t, *J* = 6.1 Hz, 1 H), 3.88 – 3.76 (m, 2 H)

^13^C NMR (CDCl_3_, 100 MHz): δ= 199.01, 171.02, 136.54, 134.01, 133.97, 133.67, 131.53, 129.13, 128.76, 128.65, 128.60, 127.90, 127.51, 126.68, 125.87, 125.49, 123.35, 56.27, 35.41

HRMS (ESI+): *m*/*z*

[M + H]+ calcd for C_20_H_18_NO_2_: 304.1338; found: 304.1338


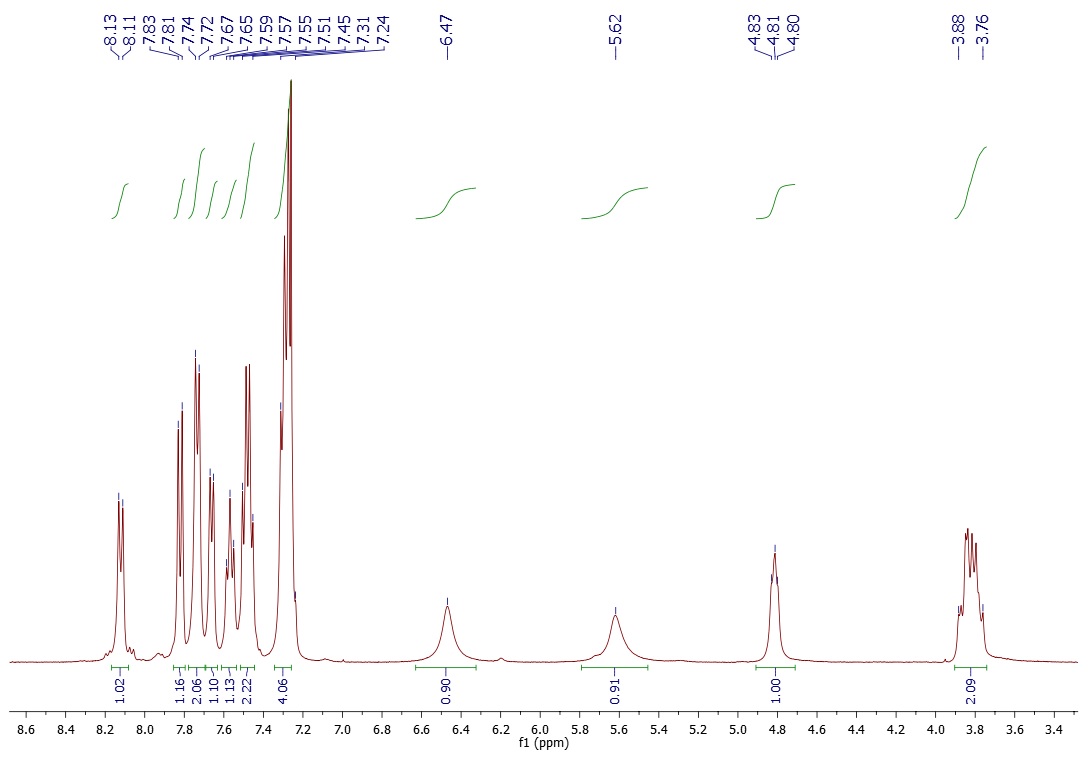


**Figure S11. ^1^H NMR of 2-(1-naphthylmethyl)-3-oxo-3-phenyl-propanamide**

**
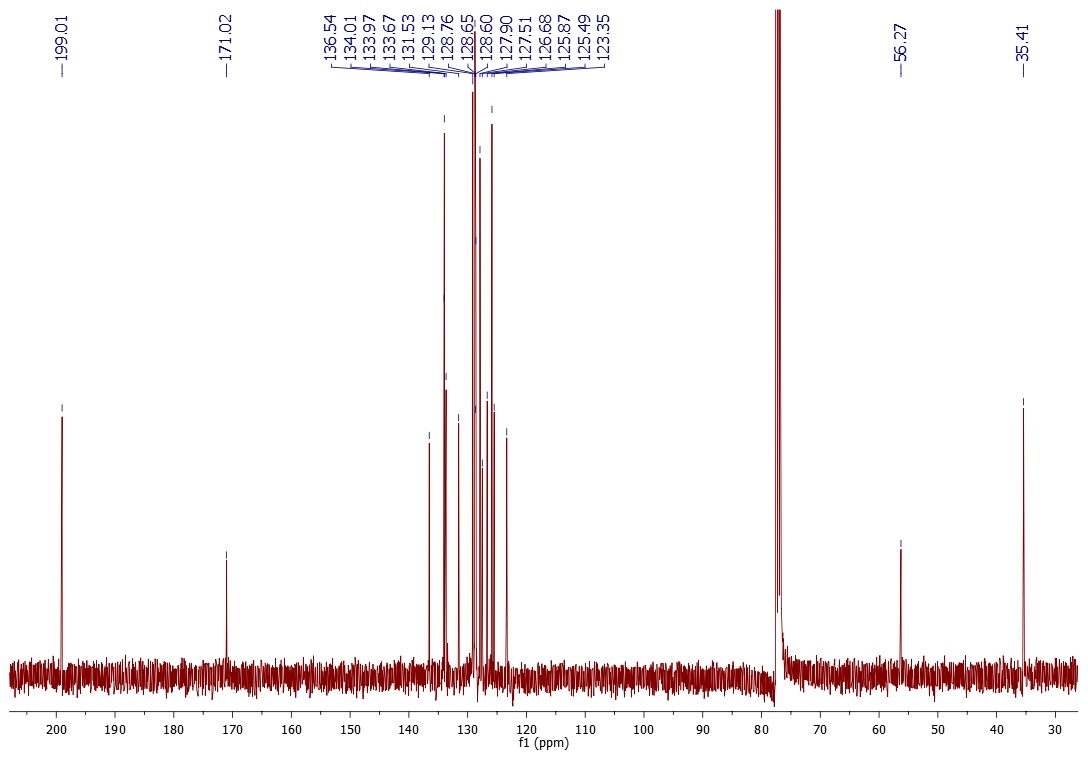
**

**Figure S12. ^13^C NMR of 2-(1-naphthylmethyl)-3-oxo-3-phenyl-propanamide**

**2. 2-(2-naphthylmethyl)-3-oxo-3-phenyl-propanamide (3b)**

White amorphous powder, yield: 31%,

^1^H NMR (CDCl_3_, 400 MHz): δ= 7.93 – 7.91 (m, 1 H), 7.77 – 7.71 (m, 3 H), 7.66 – 7.62 (m, 1 H), 7.46 – 7.38 (m, 6 H), 6.40 (s, NH), 5.74 (s, NH), 4.73 (t, *J* = 7.3 Hz, 1 H), 3.57 (dd, *J^2^* = 13.7 Hz, *J^3^* = 7.3 Hz, 1 H), 3.46 (dd, *J^2^* = 13.7 Hz, *J^3^* = 7.3 Hz, 1 H)

^13^C NMR (CDCl_3_, 100 MHz): δ= 198.61, 171.00, 136.47, 135.27, 134.13, 133.62, 132.48, 130.24, 128.96, 128.78, 128.48, 127.77, 127.73, 127.11, 126.25, 125.83, 57.57, 38.16

HRMS (ESI+): *m*/*z*

[M + H]+ calcd for C_20_H_18_NO_2_: 304.1338; found: 304.1335

**
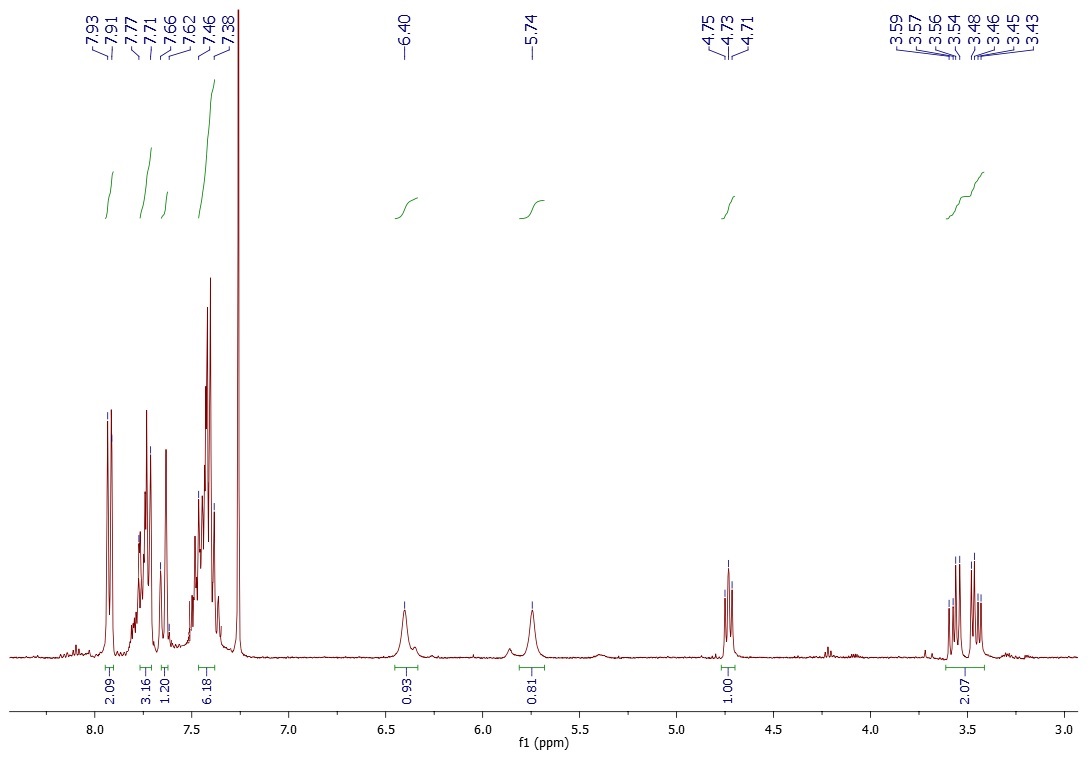
**

**Figure S13. ^1^H NMR of 2-(2-naphthylmethyl)-3-oxo-3-phenyl-propanamide**

**
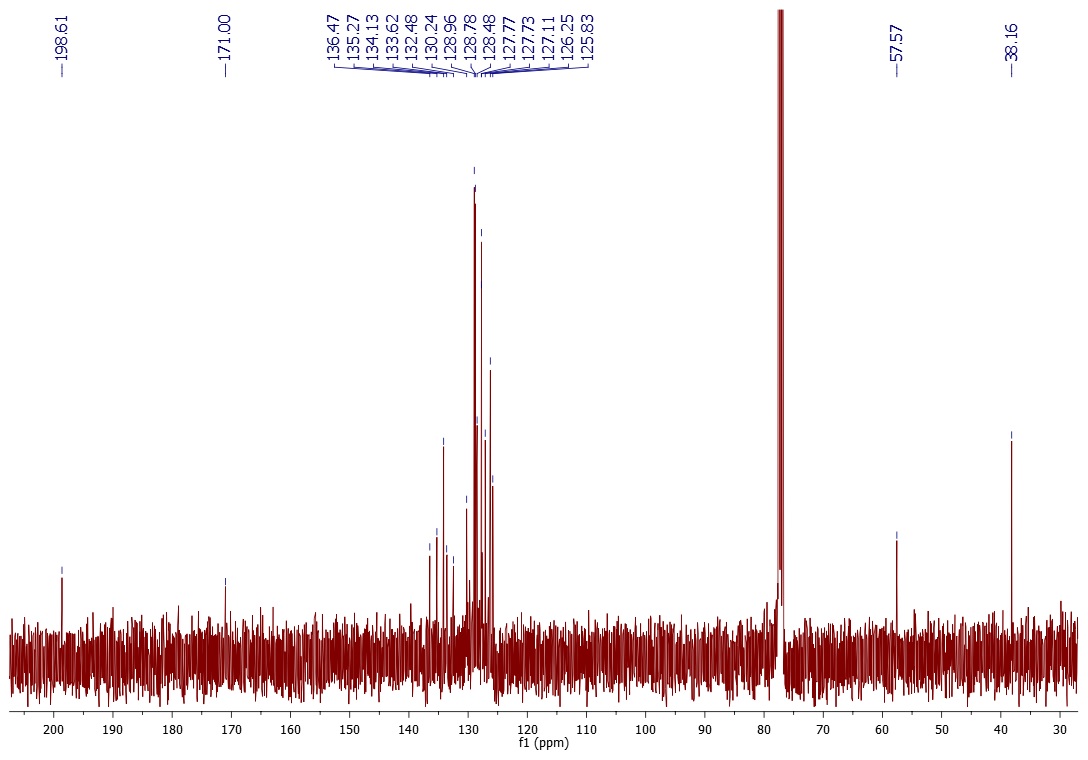
Figure S14. ^13^C NMR of 2-(2-naphthylmethyl)-3-oxo-3-phenyl-propanamide**

**3. 3-oxo-3-phenyl-2-[(4-phenylphenyl)methyl]propanamide (3c)**

White amorphous powder, yield: 51%,

^1^H NMR (DMSO, 500 MHz): δ = 8.05 – 8.03 (m, 2 H), 7.74 (s, NH), 7.65 – 7.62 (m, 3 H), 7.57 – 7.56 (m, 2 H), 7.54 – 7.51 (m, 2 H), 7.46 – 7.42 (m, 3 H), 7.37 – 7.35 (m, 2 H), 7.14 (s, NH), 4.74 (t, *J* = 7.2 Hz, 1 H), 3.23 (dd, *J^2^* = 13.9 Hz, *J^3^* = 6.9 Hz, 1 H ), 3.12 (dd, *J^2^* = 13.9 Hz, *J^3^* = 6.9Hz, 1 H)

^13^C NMR (DMSO, 125 MHz): δ= 194.96, 169.87, 139.97, 138.67, 138.01, 136.12, 133.35, 129.52, 128.88, 128.70, 128.31, 128.18, 126.48, 126.42, 56.11, 34.00

HRMS (ESI+): *m*/*z*

[M + H]+ calcd for C_22_H_20_NO_2:_ 330.1494; found: 330.1475

**
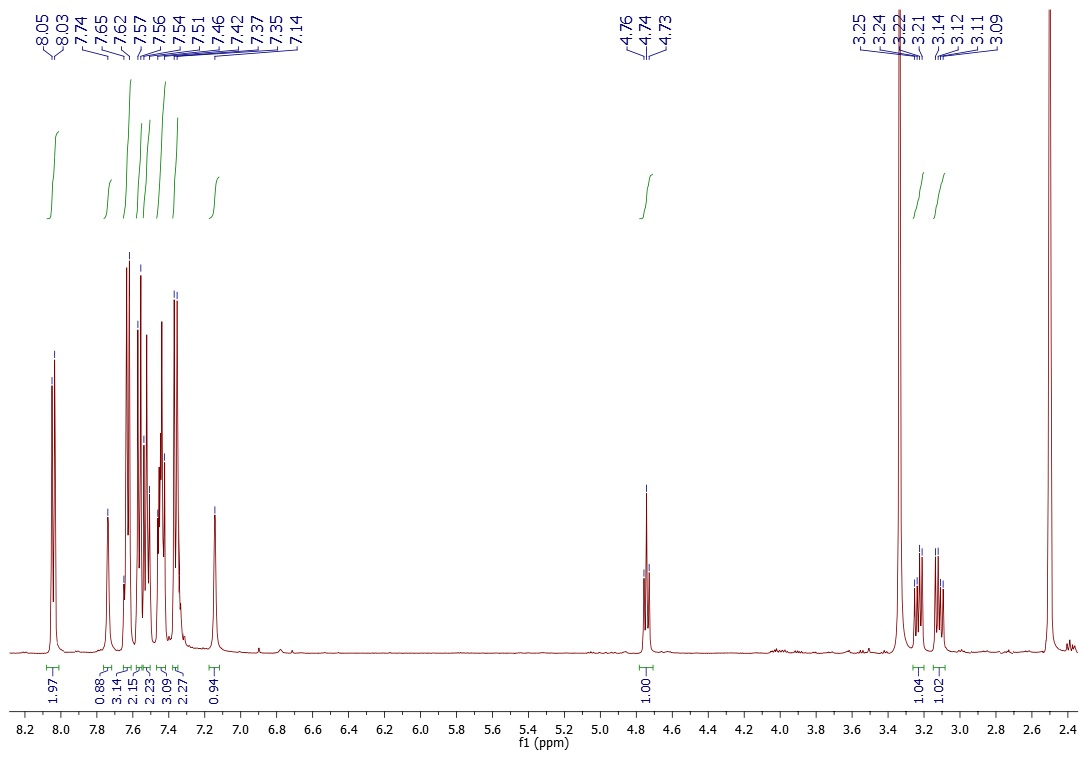
**

**Figure S15. ^1^H NMR of 3-oxo-3-phenyl-2-[(4-phenylphenyl)methyl]propanamide**


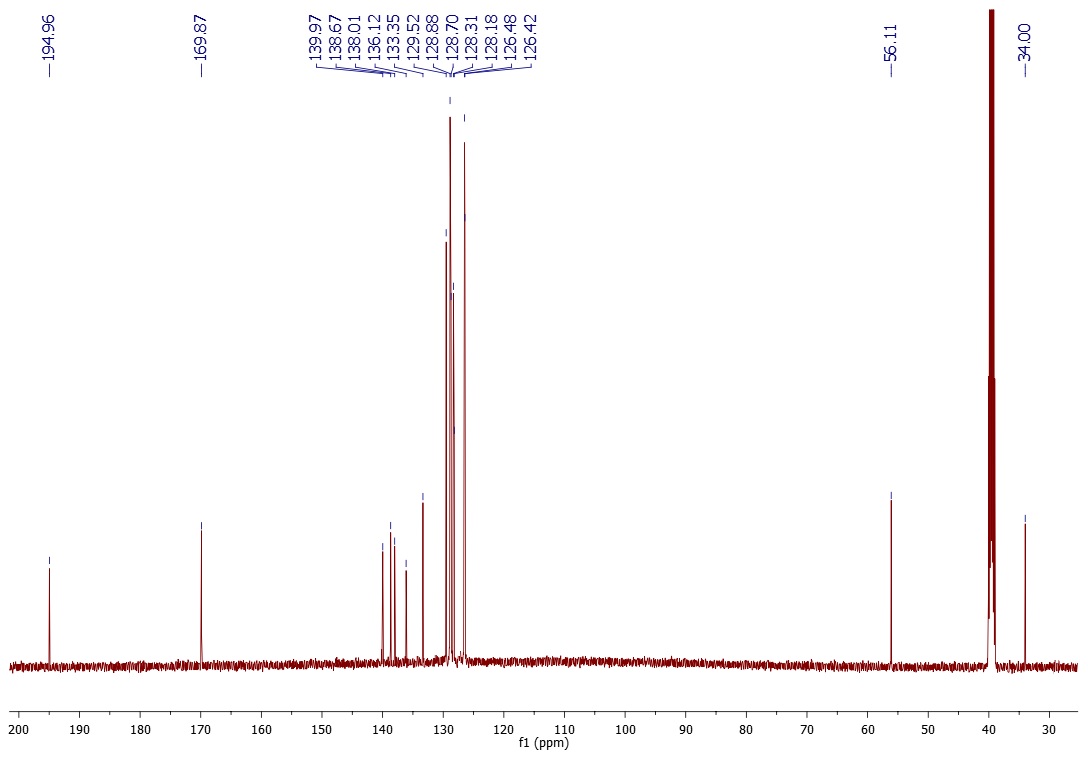


**Figure S16. ^13^C NMR of 3-oxo-3-phenyl-2-[(4-phenylphenyl)methyl]propanamide**

**4. 3-oxo-3-phenyl-2-[(3-phenylphenyl)methyl]propanamide (3d)**

White amorphous powder, yield: 50%,

^1^H NMR (CDCl_3_, 400 MHz): δ= 7.93 – 7.91 (m, 2 H), 7.59 – 7.53 (m, 2 H), 7.49 – 7.46 (m, 2 H), 7.43 – 7.38 (m, 5 H), 7.34 – 7.27 (m, 2 H), 7.17 – 7.15 (d, *J* = 7.6, 1 H), 6.59 (s, NH), 6.11 (s, NH), 4.69 (dd, *J^3^_a_* = 6.4 Hz, *J^3^_b_*= 8.3 Hz*,* 1 H), 3.45 (dd, *J^2^* = 13.7 Hz, *J^3^* = 8.3 Hz, 1 H), 3.37 (dd, *J^2^* = 13.7 Hz, *J^3^* = 6.4 Hz, 1 H)

^13^C NMR (CDCl_3_, 100 MHz): δ= 198.58, 171.45, 141.62, 140.95, 138.18, 136.46, 134.12, 129.15, 128.94, 128.84, 128.78, 127.87, 127.86, 127.46, 127.22, 125.89, 57.30, 38.18

HRMS (ESI+): *m*/*z*

[M + H]+ calcd for C_22_H_20_NO_2_: 330.1494; found: 330.1492


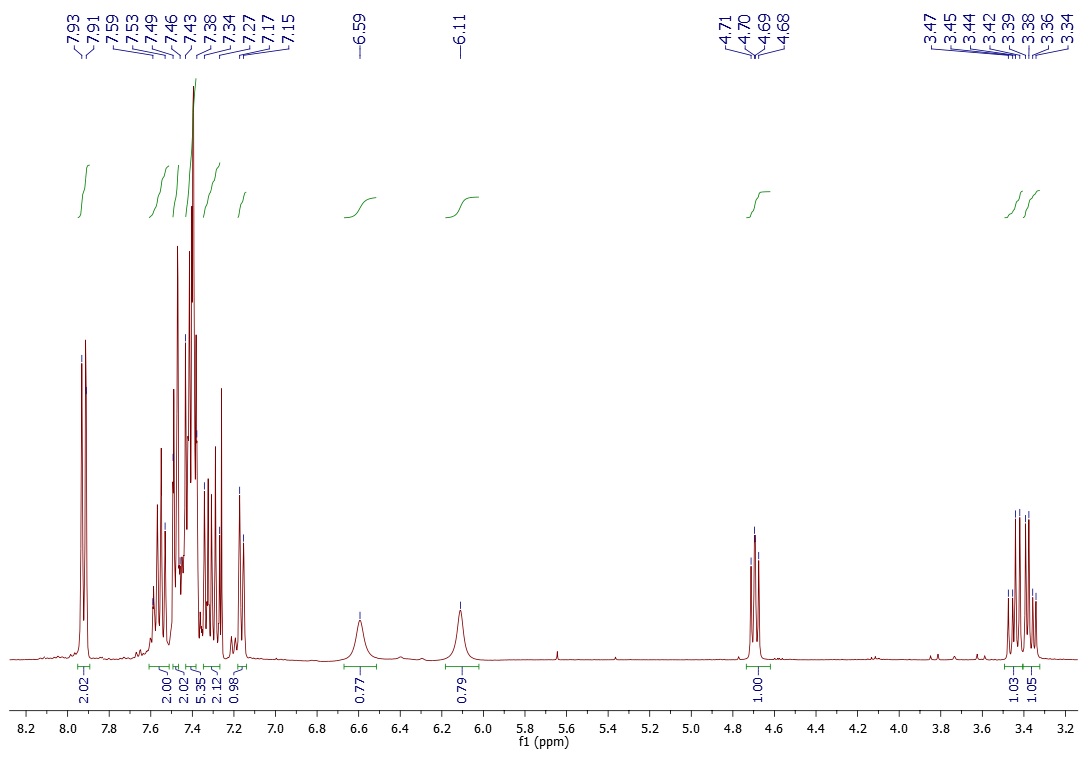


**Figure S17. ^1^H NMR of ethyl 3-oxo-3-phenyl-2-[(3-phenylphenyl)methyl]propanamide**


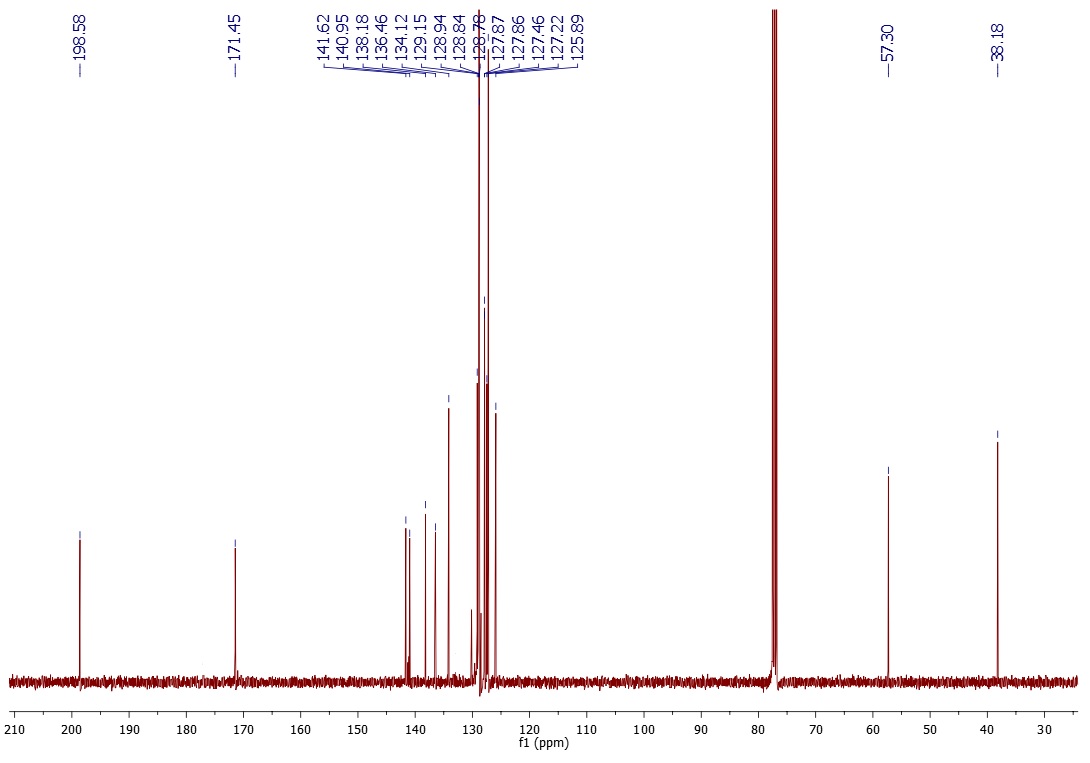


**Figure S18. ^13^C NMR of ethyl 3-oxo-3-phenyl-2-[(3-phenylphenyl)methyl]propanamide**

**5. 3-oxo-3-phenyl-2-[[4-(trifluoromethyl)phenyl]methyl]propanamide (3e)**

White amorphous powder, yield: 80%,

^1^H NMR (CDCl_3_, 400 MHz): δ= 7.93 – 7.90 (m, 1 H), 7.61 – 7.54 (m, 2 H), 7.50-7.43 (m, 3 H), 7.34 – 7.29 (m, 3 H), 6.36 (s, NH), 5.78 (s, NH), 4.62 (dd, *J^3^_a_* = 8.2 Hz, *J^3^_b_* = 6.6 Hz, 1 H), 3.44 (dd, *J^2^* = 13.7 Hz, *J^3^* = 8.2 Hz, 1 H), 3.44 (dd, *J^2^* = 13.7 Hz, *J^3^* = 6.6 Hz, 1 H),

^13^C NMR (CDCl_3_, 100 MHz): δ= 198.17, 176.25, 144.40**,** 136.21, 134.41, 129.40, 129.10, 129.00 (q, *J^2^* = 24.8 Hz), 128.80, 125.67 (q, *J^3^* = 3.3 Hz), 57.18, 30.51

HRMS (ESI+): *m*/*z*

[M + H]+ calcd for C_17_H_15_F_3_NO_2_: 322.1055; found: 322.1061


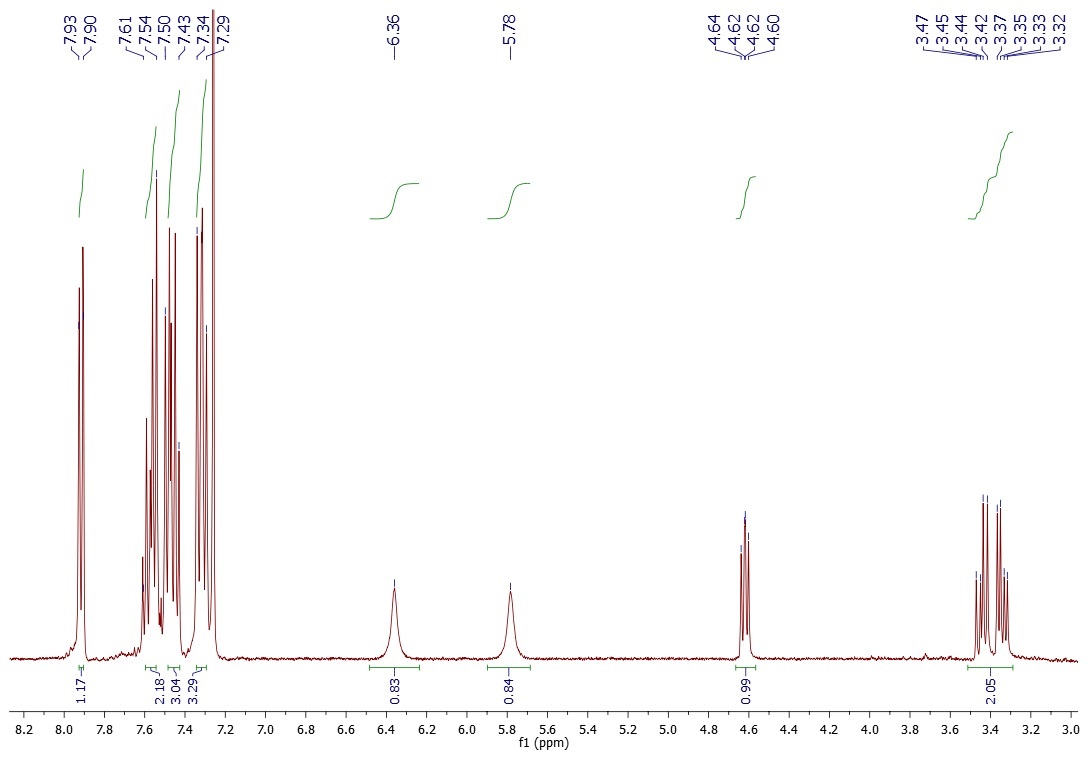


**Figure S19. ^1^H NMR of 3-oxo-3-phenyl-2-[[4-(trifluoromethyl)phenyl]methyl]propanamide**


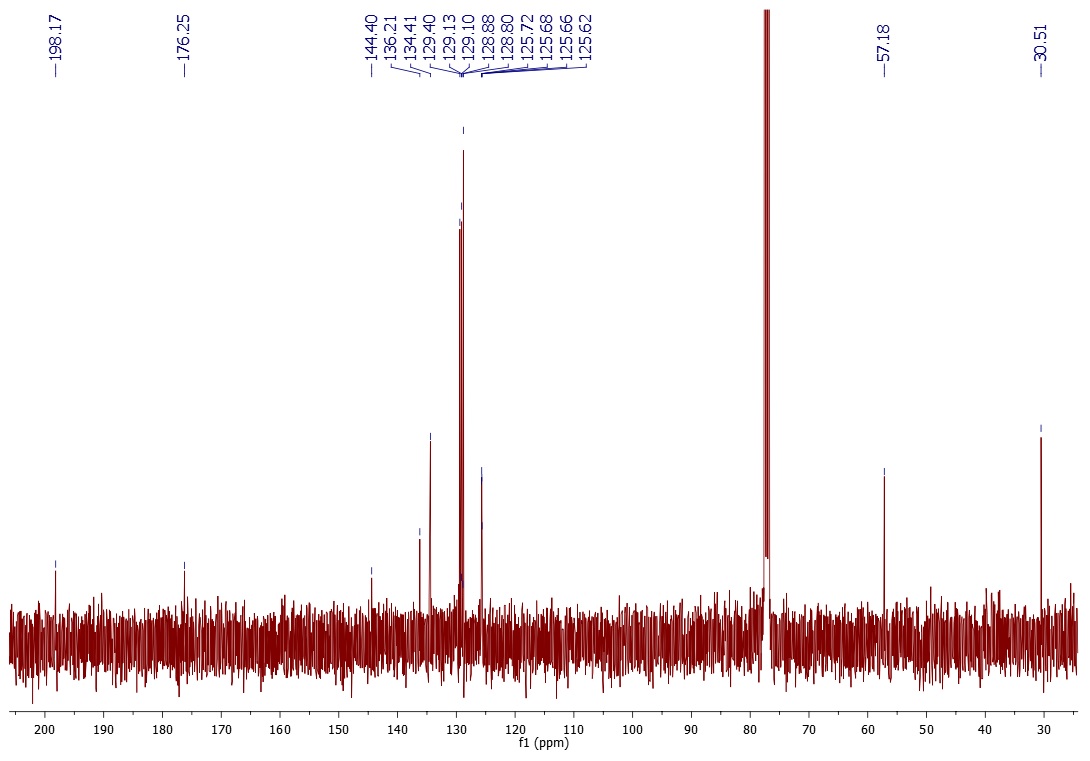


**Figure S20. ^13^C NMR of 3-oxo-3-phenyl-2-[[4-(trifluoromethyl)phenyl]methyl]propanamide**

**C. General procedure for benzyl-type derivatives of 4-phenyl-5,6,7,8-tetrahydro-1H-quinolin-2-one [1]**

A mixture of 3-oxo-3-phenyl-propanamide derivatives **3a-e** (0.19 mmol), cyclohexanone (0.28 mmol), TsOH (0.38 mmol), and 2.5 ml of toluene was added to round-bottomed flask fitted with Soxhlet apparatus with MgSO_4_ and stir bar. Reaction mixture was heated to reflux and left overnight. The solvent was evaporated. Residue was dissolved in AcOEt and washed with water, 2M NaOH and brine. Organic layer was dried with anhydrous MgSO_4._ The titled products were isolated by flash column chromatography (dichloromethane/methanol 200:1). Yields and characteristics of title compounds are collected below.

**1. 3-(1-naphthylmethyl)-4-phenyl-5,6,7,8-tetrahydro-1H-quinolin-2-one (4a)**

White amorphous powder, yield: 75%

Mixture of tautomers 9:1

^1^H NMR (CDCl_3_, 400 MHz): δ= 7.86 (d, *J* = 8.2 Hz, 1 H), 7.79 – 7.76 (m, 1 H), 7.62 (d, *J* = 8.12 Hz, 1 H), 7.42 – 7.35 (m, 2 H), 7.23 – 7.09 (m, 4 H + 0.8 NH), 6.92 – 6.87 (m, 3 H), 5.30 (s, 0.2 OH), 4.13 (s, 1.8 H), 3.93 (s, 0.2 H), 2.84 (t, *J* = 6.5 Hz, 0.2 H), 2.67 (t, *J* = 5.9 Hz, 1.74 H), 2.21 (t, *J* = 6.5 Hz, 0.26 H), 2.05 (t, *J* = 5.9 Hz, 1.8 H), 1.75 – 1.69 (m, 2 H), 1.64 – 1.59 (m, 2 H)

^13^C NMR (CDCl_3_, 100 MHz): δ= 162..17, 157.23, 136.04, 133.69, 132.02, 128.63, 128.56, 127.95, 127.27, 126.51, 125.71, 125.43, 125.39, 124.91, 123.80, 117.68, 29.62, 27.32, 26.03, 22.63, 21.47

HRMS (ESI+): *m*/*z*

[M + H]+ calcd for C_26_H_24_NO: 366.1857; found: 366.1862.


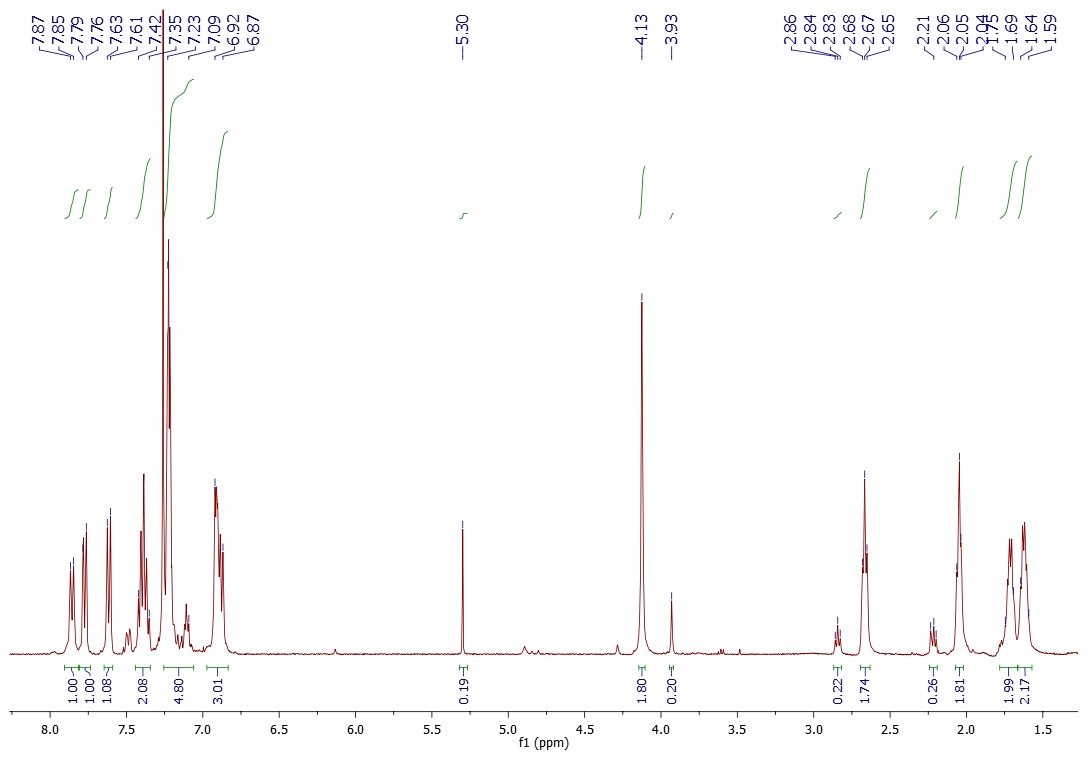


**Figure S21. ^1^H NMR of 3-(1-naphthylmethyl)-4-phenyl-5,6,7,8-tetrahydro-1H-quinolin-2-one**


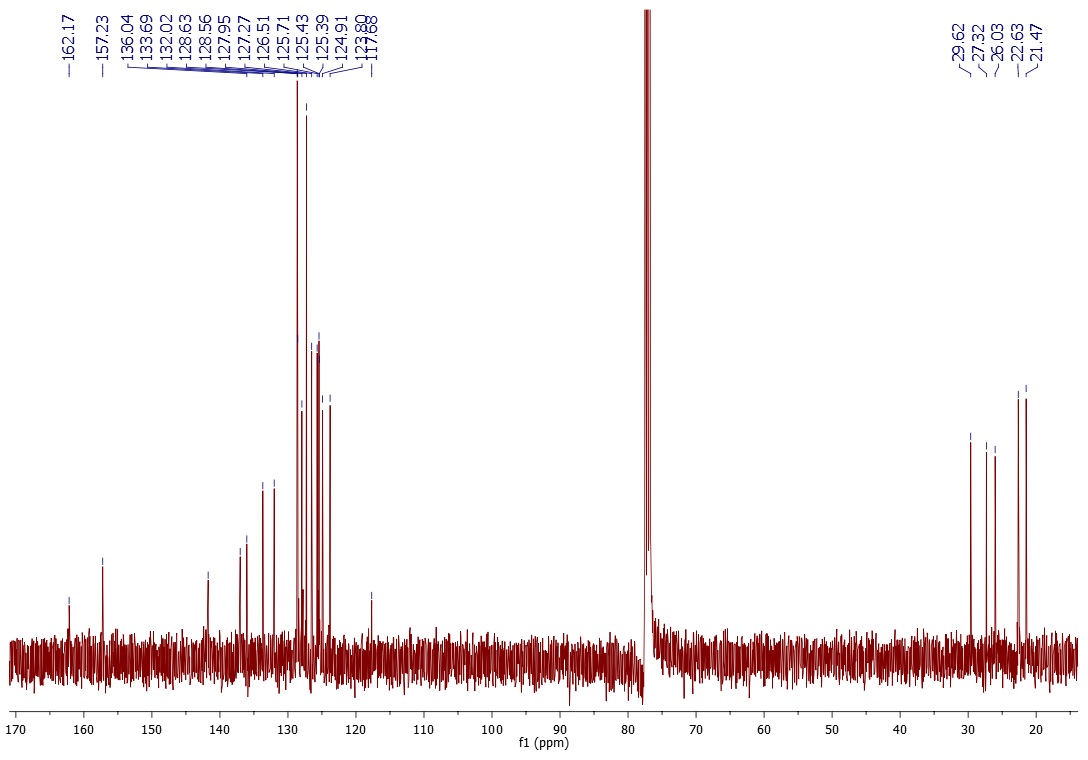


**Figure S22. ^13^C NMR of of 3-(1-naphthylmethyl)-4-phenyl-5,6,7,8-tetrahydro-1H-quinolin-2-one**

**2. 3-(2-naphthylmethyl)-4-phenyl-5,6,7,8-tetrahydro-1H-quinolin-2-one (4b)**

Bright yellow amorphous powder, yield: 85%

Mixture of tautomers 4:1

^1^H NMR (CDCl_3_, 400 MHz): δ= 7.83 – 7.66 (m, 3 H), 7.66 – 7.60 (m, 1.6 H), 7.47 – 7.42 (m, 1.64 H), 7.39 – 7.34 (m, 4.36 H), 7.22 (s, 0.9 NH), 6.97 – 6.95 (m, 1.4 H), 5.29 (s, 0.1 OH), 3.86 (s, 1.78 H), 3.73 (s, 0.22 H), 3.14 (t, *J* = 7.7 Hz, 0.44 H), 2.73 (t, *J* = 6.1 Hz, 1.56 H), 2.62 (t, *J* = 7.7 Hz, 0.43 H), 2.05 (t, *J* = 6.1 Hz, 1.57), 1.71 – 1.65 (m, 2 H), 1.62 – 1.57 (m, 2 H)

^13^C NMR (CDCl_3_, 100 MHz): δ= 161.93, 157.19, 133.46, 132.09, 128.74, 128.20, 127.77, 127.68, 127.66, 127.58, 127.47, 127.44, 126.80, 125.79, 125.22, 118.62, 29.49, 27.29, 26.01, 22.50, 21.33

HRMS (ESI+): *m*/*z*

[M + H]+ calcd for C_26_H_24_NO: 366.1857; found: 366.1856


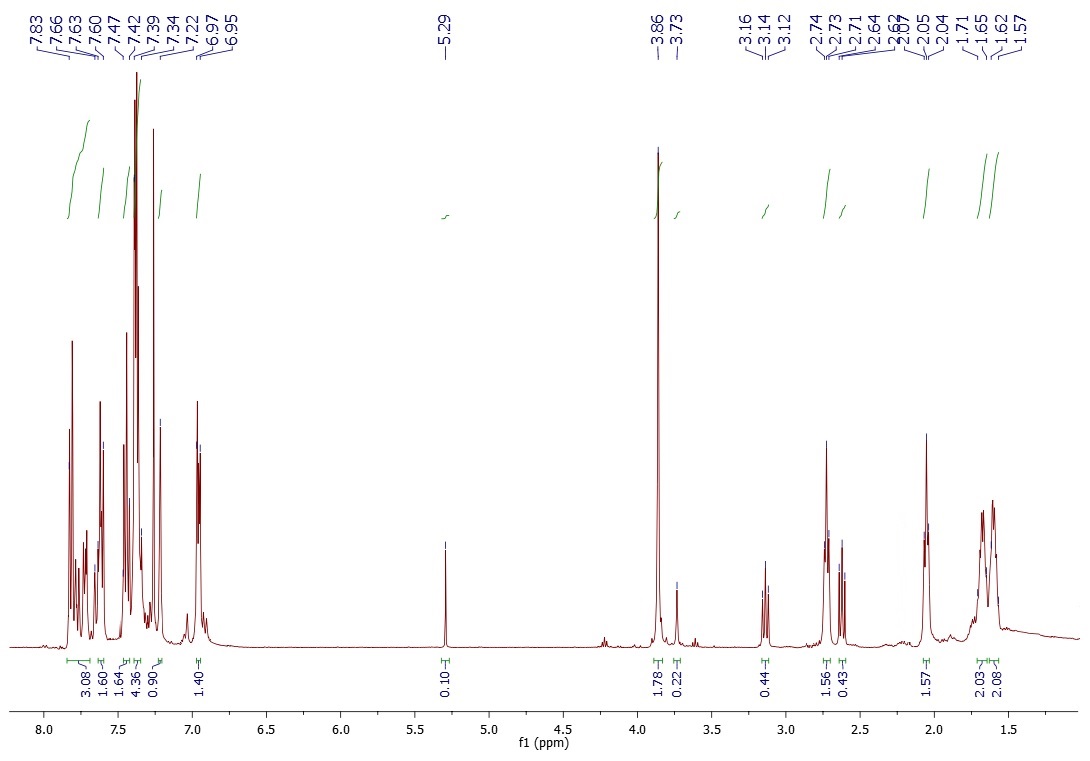


**Figure S23. ^1^H NMR of 3-(2-naphthylmethyl)-4-phenyl-5,6,7,8-tetrahydro-1H-quinolin-2-one**

**
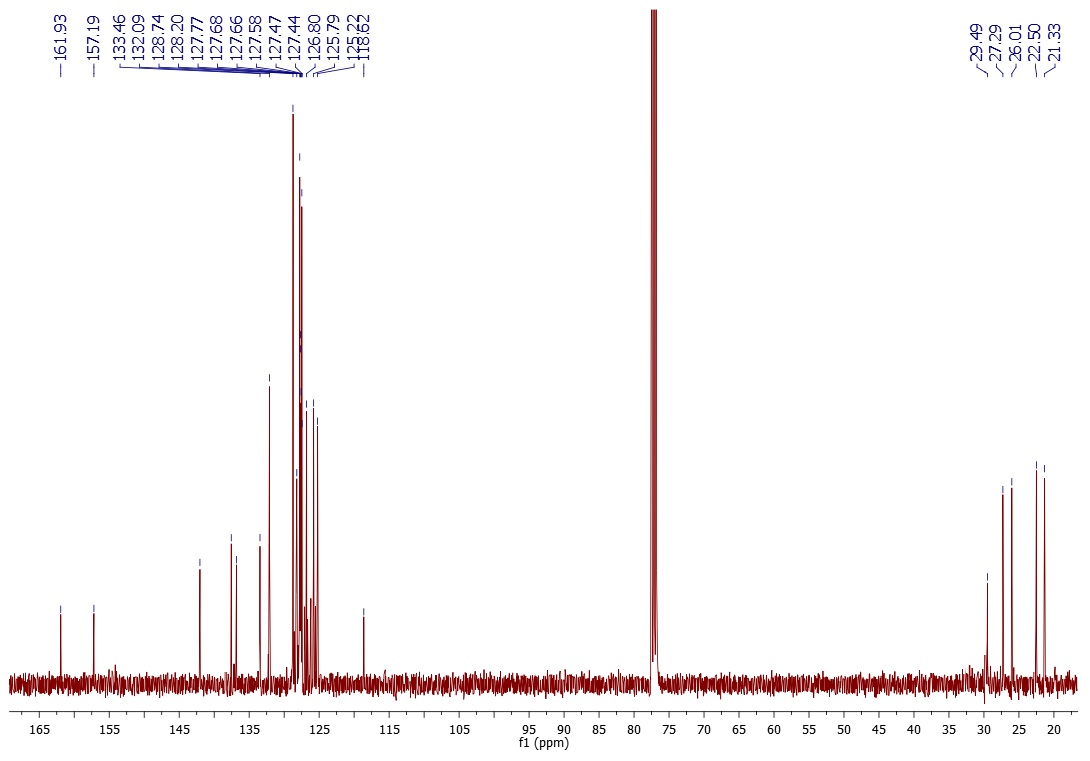
**

**Figure S24. ^13^C NMR of 3-(2-naphthylmethyl)-4-phenyl-5,6,7,8-tetrahydro-1H-quinolin-2-one**

**3. 4-phenyl-3-[(4-phenylphenyl)methyl]-5,6,7,8-tetrahydro-1H-quinolin-2-one (4c)**

White amorphous powder, yield: 33%

Mixture of rotamers 9:1

^1^H NMR (CDCl_3_, 400 MHz): δ= 7.83 – 7.81 (m, 1 H), 7.54 – 7.51 (m, 3 H), 7.49 – 7.46 (m, 4 H), 7.43 – 7.36 (m, 5 H), 7.34 – 7.32 (m, 1 H), 7.01 – 6.99 (m, 1 H), 3.82 (s, 0.26 H), 3.79 (s, 1.74 H), 3.00 (t, *J* = 7.7 Hz, 0.23 H), 2.97 (t, *J* = 6.2 Hz, 1.75 H), 2.59 (t, *J* = 7.7 Hz, 0.25 H), 2.22 (t, *J* = 6..2 Hz, 1.77 H), 1.89 – 1.83 (m, 2 H), 1.74 – 1.68 (m, 2 H)

^13^C NMR (CDCl_3_, 100 MHz): δ= 160.71, 159.14, 140.83, 139.60, 137.18, 135.17, 132.24, 129.21, 129.01, 128.88, 128.79, 127.51, 127.38, 127.18, 127.10, 115.45, 31.61, 27.67, 26.24, 22.07, 21.00

HRMS (ESI+): *m*/*z*

[M + H]+ calcd for C_28_H_26_NO: 392.2014; found: 392.2018


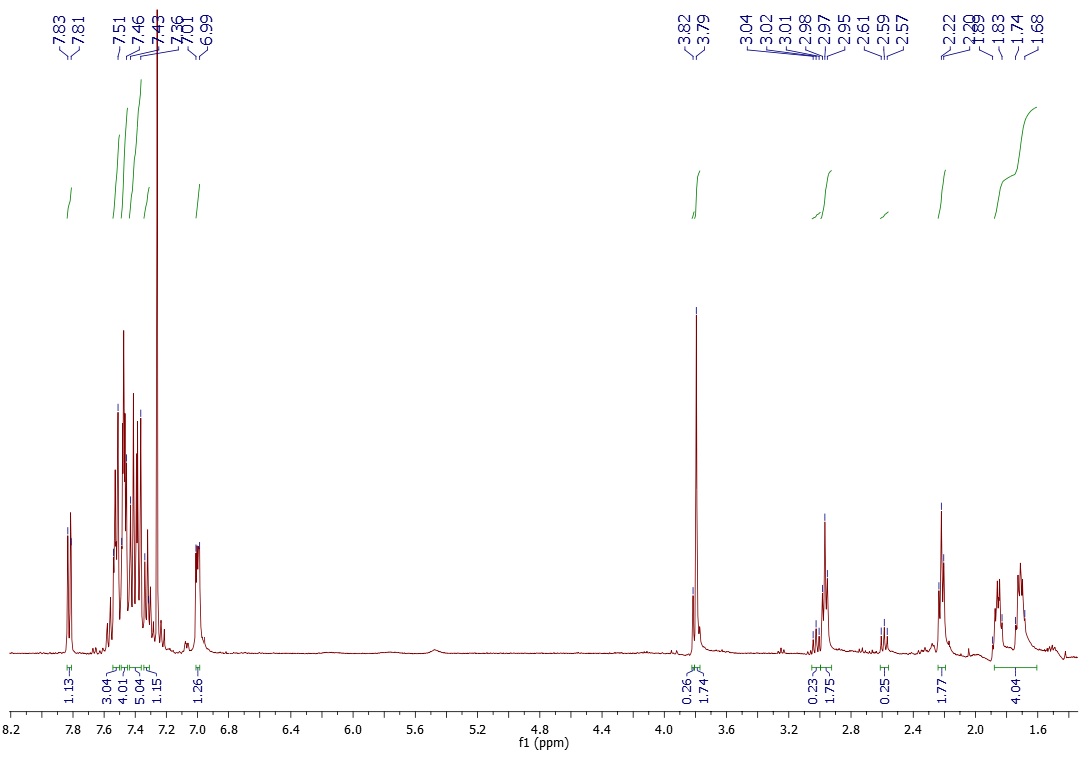


**Figure S25. ^1^H NMR of 4-phenyl-3-[(4-phenylphenyl)methyl]-5,6,7,8-tetrahydro-1H-quinolin-2-one**


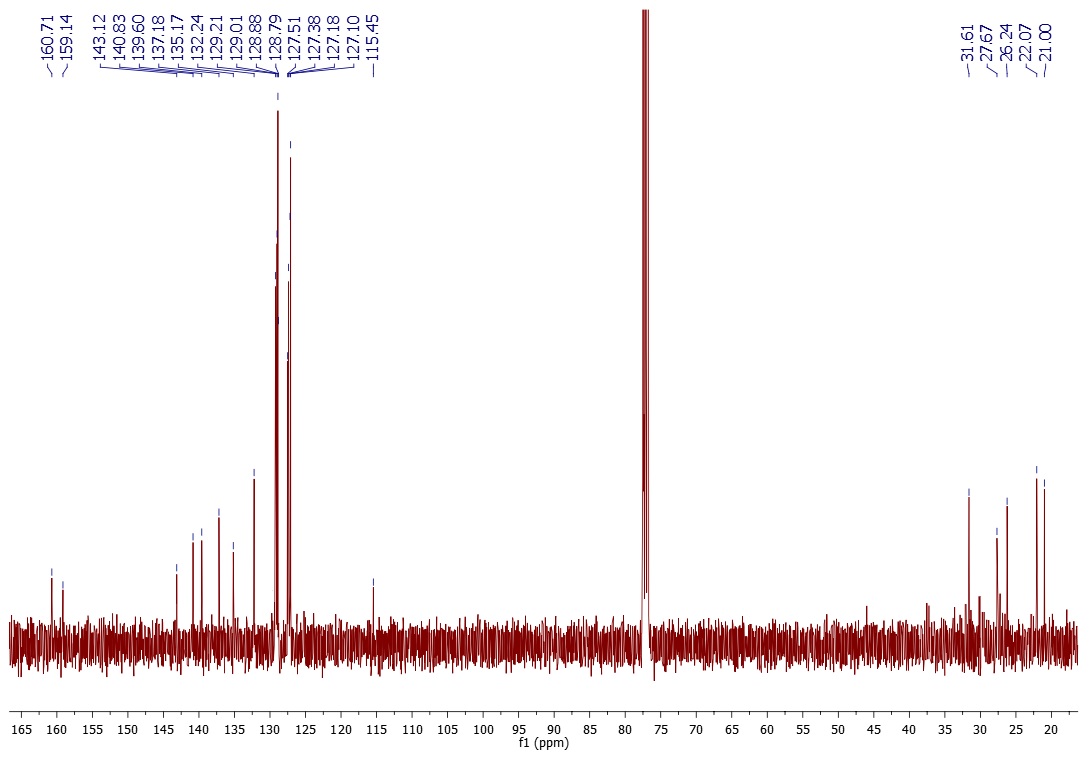


**Figure S26. ^13^C NMR of 4-phenyl-3-[(4-phenylphenyl)methyl]-5,6,7,8-tetrahydro-1H-quinolin-2-one**

**4. 4-phenyl-3-[(3-phenylphenyl)methyl]-5,6,7,8-tetrahydro-1H-quinolin-2-one (4d)**

White powder, yield: 40%

Mixture of rotamers 9:1

^1^H NMR (CDCl_3_, 400 MHz): δ= 7.484 – 7.81 (m, 0.26 H), 7.51 – 7.47 (m, 2 H), 7.45 – 7.33 (m, 5 H + 0.95 NH), 7.32 – 7.28 (m, 2 H), 7.22 – 7.16 (m, 2 H), 7.02 – 6.99 (m, 2.74 H), 5.29 (s, 0.05 OH), 3.77 (s, 1.89 H), 3.71 (s, 0.11 H), 3.05 (t, *J* = 7.8 Hz, 0.14 H), 2.69 (t, *J* = 6.1 Hz, 1.8 H), 2.59 (t, *J* = 7.8 Hz, 0.2 H), 2.03 (t, *J* = 6.1 Hz, 1.76 H), 1.71 – 1.66 (m, 2 H), 1.62 – 1.58 (m, 2 H)

^13^C NMR (CDCl_3_, 100 MHz): δ= 162.57, 156.21, 141.71, 141.41, 140.94, 140.82, 137.26, 128.73, 128.70, 127.91, 127.18, 127.15, 117.26, 32.86, 27.19, 26.01, 22.63, 21.48

HRMS (ESI+): *m*/*z*

[M + H]+ calcd for C_28_H_26_NO: 392.2014; found: 392.2030.

**
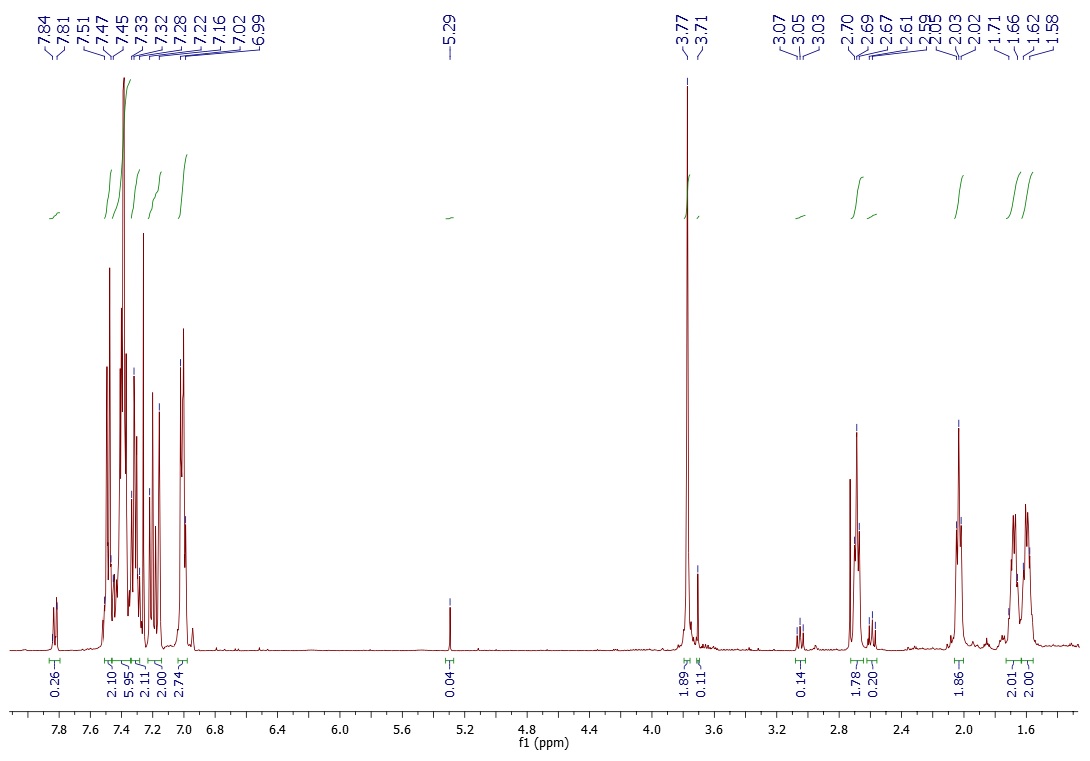
**

**Figure S27. ^1^H NMR of 4-phenyl-3-[(3-phenylphenyl)methyl]-5,6,7,8-tetrahydro-1H-quinolin-2-one**

**
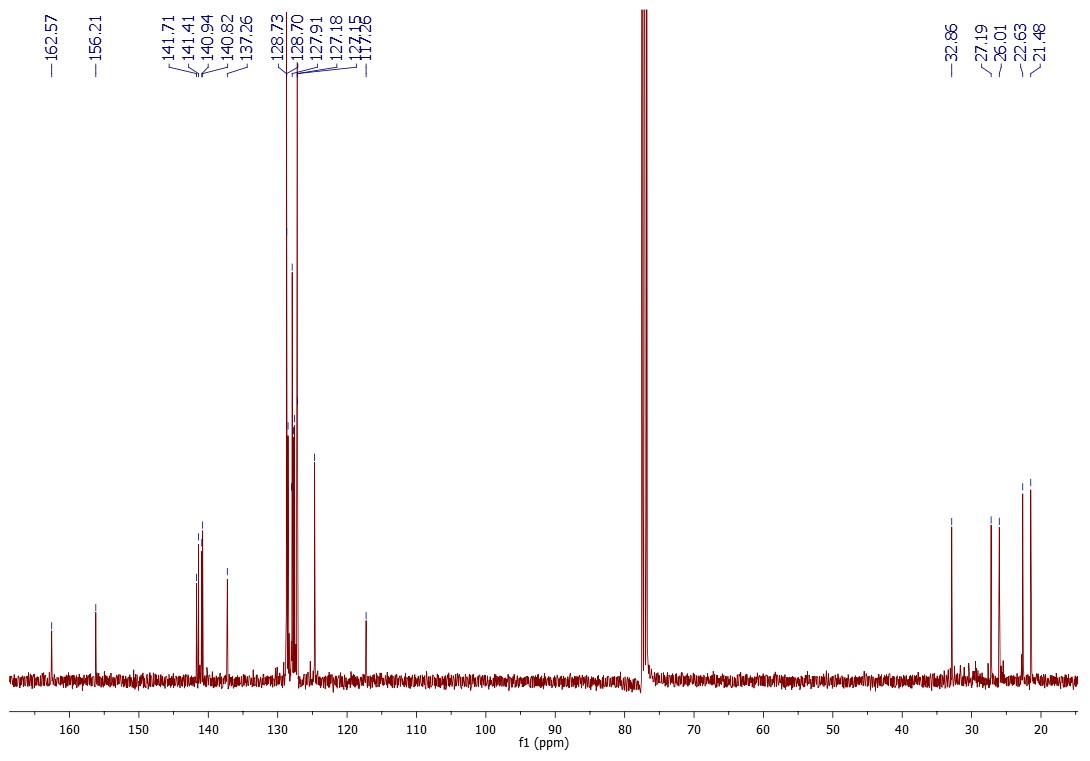
**

**Figure S28. ^13^C NMR of 4-phenyl-3-[(3-phenylphenyl)methyl]-5,6,7,8-tetrahydro-1H-quinolin-2-one**

**5. 4-phenyl-3-[[4-(trifluoromethyl)phenyl]methyl]-5,6,7,8-tetrahydro-1H-quinolin-2-one (4e)**

White powder, yield: 39%,

Mixture of rotamers 9:1

^1^H NMR (CDCl_3_, 400 MHz): δ= 7.83 – 7.81 (m, 0.5 H), 7.47 – 7.34 (m, 6 H), 7.06 – 7.04 (d, *J* = 8 Hz, 2 H), 6.98 – 6.95 (m, 1.5 H), 3.74 (s, 2 H), 3.03 (t, *J* = 7.6 Hz, 0.18 H), 2.72 (t, *J* = 6.2 Hz, 1.8 H), 2.55 (t, *J* = 7.6 Hz, 0.2 H), 2.06 (t, *J* = 6.2 Hz, 1.82 H), 1.77 – 1.71 (m, 2 H), 1.65 – 1.60 (m, 2 H)

^13^C NMR (CDCl_3_, 100 MHz): δ= 162.18, 156.84, 144.42 (q, *J^5^* = 1.3 Hz), 142.26, 136.87, 132.12, 128.93, 128.87, 128.75, 128.26 (q, *J^2^* = 25.7 Hz), 127.66, 124.99 (q, *J^3^* = 3.7 Hz), 124.44 (q, *J^1^* = 270 Hz), 117.90, 32.71, 27.27, 25.99, 22.55, 21.39

HRMS (ESI+): *m*/*z*

[M + H]+ calcd for C_23_H_21_F_3_NO: 384.1575; found: 384.1603.


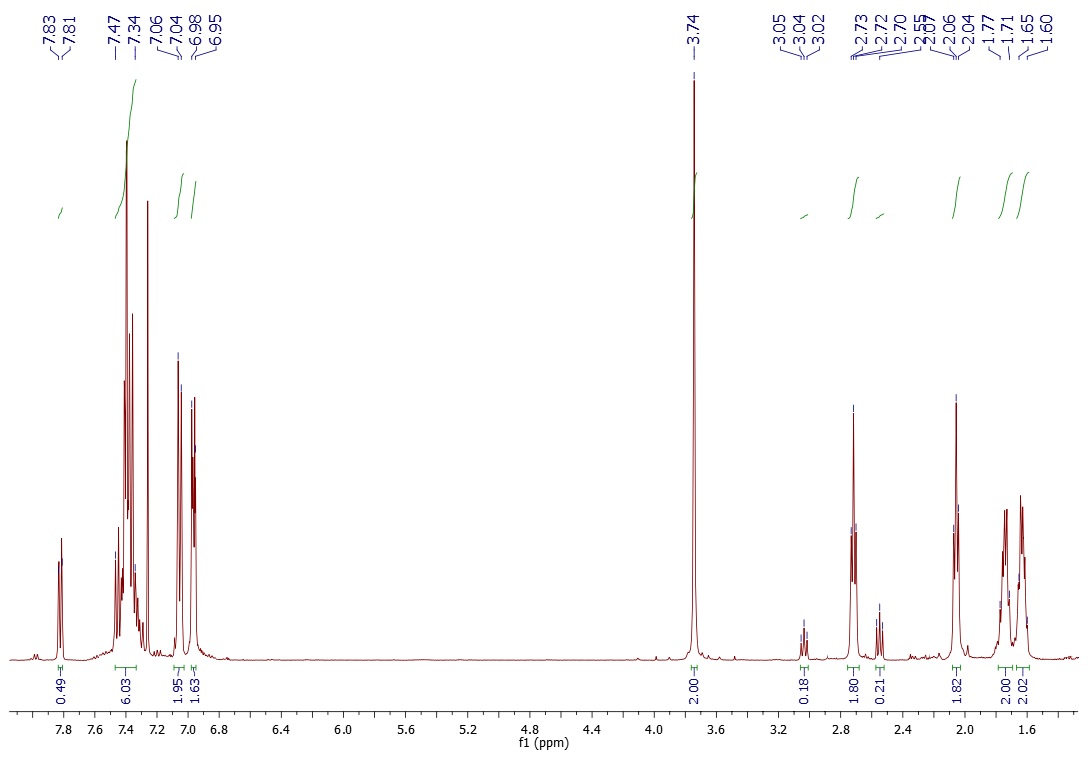


**Figure S29. ^1^H NMR of 4-phenyl-3-[[4-(trifluoromethyl)phenyl]methyl]-5,6,7,8-tetrahydro-1H-quinolin-2-one**


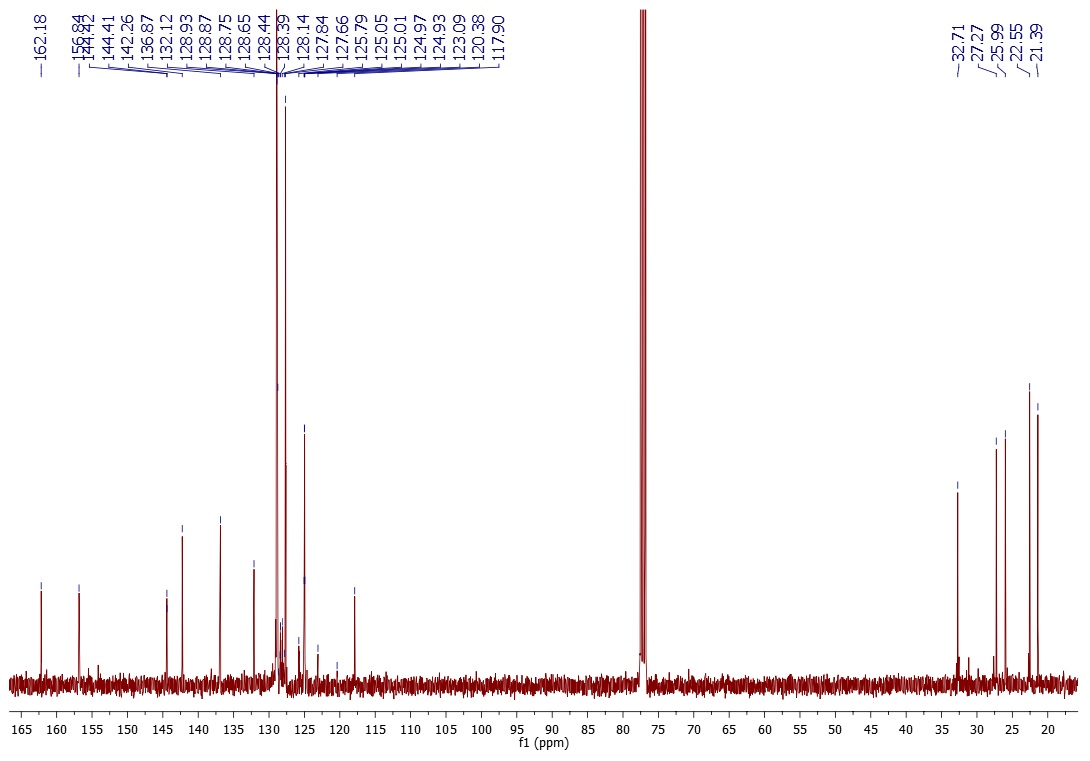


**Figure S30. ^13^C NMR of 4-phenyl-3-[[4-(trifluoromethyl)phenyl]methyl]-5,6,7,8-tetrahydro-1H-quinolin-2-one**

**6. 2-chloro-3-(1-naphthylmethyl)-4-phenyl-5,6,7,8-tetrahydroquinoline (5) [2]**

**4a** (0.13 g, 0.36 mmol) and 2 ml of PhPOCl_2_ were placed round-bottomed flask with stir bar. The reaction temperature was raised to 160^O^C and left overnight. Post-reaction mixture was gently diluted with water and washed with DCM. Organic layer was dried with anhydrous MgSO_4_. After solvent evaporation the crude product was isolated by flash column chromatography (ethyl acetate : hexane 1:25). The title compound (0.074 g, 0.19 mmol, 53%) was obtained as bright yellow powder

Bright yellow amorphous powder, yield: 53%

^1^H NMR (CDCl_3_, 400 MHz): δ= 7.83 – 7.81 (m, 2 H), 7.68 (d, *J* = 8.24 Hz, 1 H), 7.47 – 7.40 (m, 2 H), 7.31 (t, *J* = 7.68 Hz, 1 H), 7.22 – 7.18 (m, 3 H), 6.97 – 6.93 (m, 2 H), 6.81 (d, *J* = 7.12 Hz, 1 H), 4.25 (s, 2 H), 3.04 (t, *J* = 6.46, 2 H), 2.31 (t, *J* = 6.36 Hz, 2 H), 1.92 – 1.86 (m, 2 H), 1.75 – 1.69 (m, 2 H)

^13^C NMR (CDCl_3_, 100 MHz): δ= 156.49, 149.49, 137.32, 135.13, 133.64, 131.82, 130.47, 129.31 128.77, 128.69, 127.94, 127.55, 126.81, 125.96, 125.62, 125.49, 124.60, 123.12, 33.12, 29.85, 27.77, 22.77, 22.65

HRMS (ESI+): *m*/*z*

[M + H]+ calcd for C_26_H_23_ClN: 384.1519; found: 384.1555.


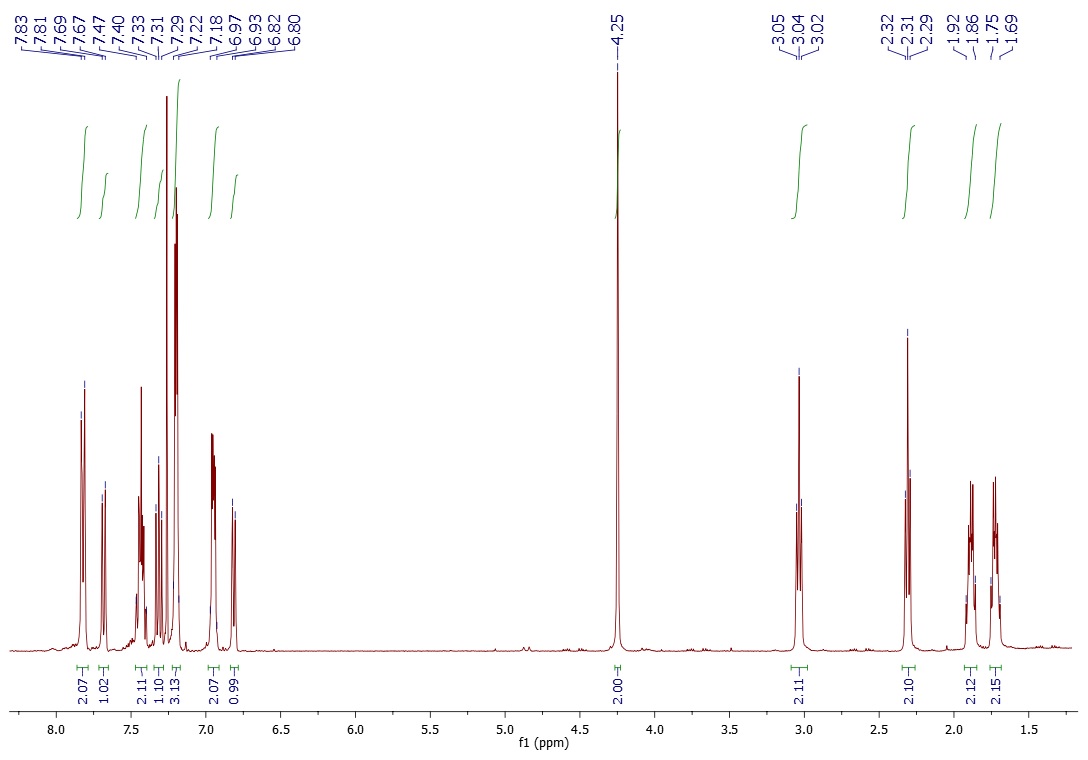


**Figure S31. ^1^H NMR of 2-chloro-3-(1-naphthylmethyl)-4-phenyl-5,6,7,8-tetrahydroquinoline**


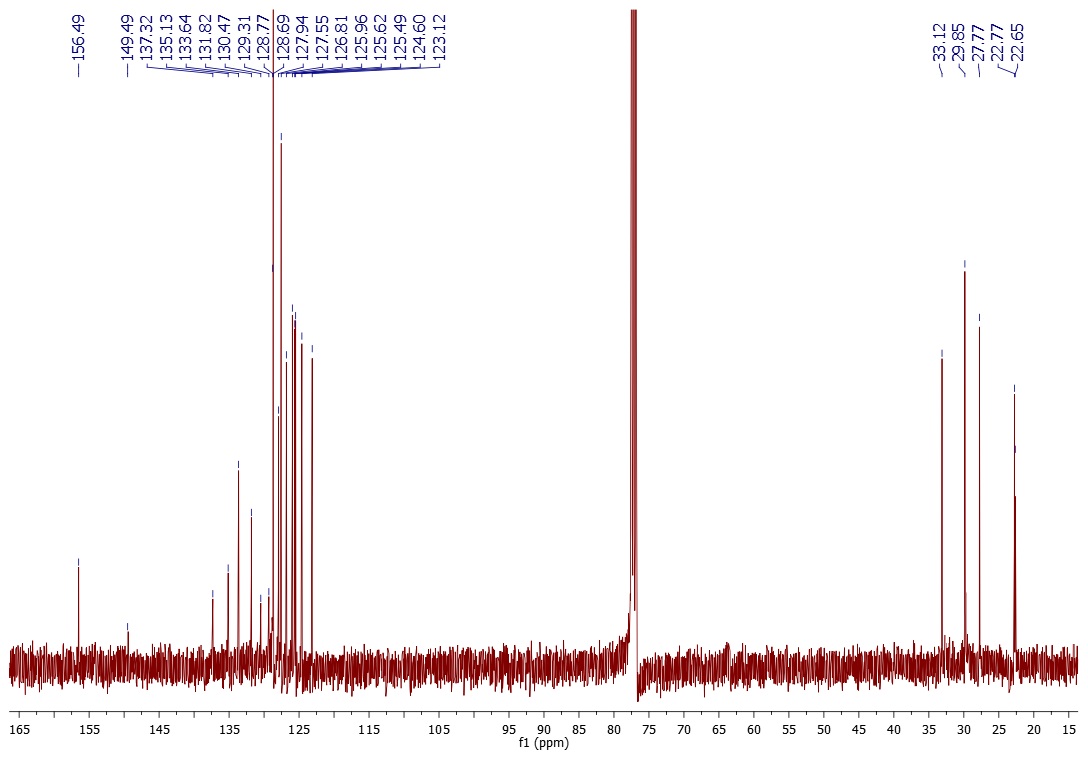


**Figure S32. ^13^C NMR of 2-chloro-3-(1-naphthylmethyl)-4-phenyl-5,6,7,8-tetrahydroquinoline**

**7. 2-methoxy-3-(1-naphthylmethyl)-4-phenyl-5,6,7,8-tetrahydroquinoline (6) [3]**

A solution of **4a** (0.105 g, 0.28 mmol) in 0.35 ml of EtOH and 9.3 ml of ACN was placed in round bottomed flask with stir bar. Ag_2_CO_3_ (0.772 g, 2.8 mmol) and MeI (0.53 ml, 8.4 mmol) were added. The mixture was left overnight in RT. Ag_2_CO_3_ was filtered by celite and solvents were evaporated. Crude product was purified by flash column chromatography (ethyl acetate : hexane 1:50). Compound **6** (0.070 g, 0.18 mmol, 66%) was obtained as white amorphous powder.

White amorphous powder, yield: 66 %,

Mixture of rotamers 9:1

^1^H NMR (CDCl_3_, 400 MHz): δ= 8.27 (dd, *J_1_* = 8.44 Hz, *J_2_* = 0.66 Hz, 0.12 H), 7.92 – 7.87 (m, 1 H), 7.82 – 7.80 (m, 0.82 H), 7.66 (d, *J* = 8.2 Hz, 0.87 H), 7.57 – 7.52 (m, 0.18 H), 7.49 – 7.36 (m, 2H), 7.32 – 7.27 (m, 1 H), 7.22 – 7.19 (m, 3 H), 7.00 – 6.95 (m, 2 H), 6.84 (dd, *J_1_* = 7.12 Hz, *J_2_* = 0.8 Hz, 0.88 H), 6.74 (d, *J* = 7.76 Hz, 0.13 H), 4.12 (s, 1.69 H), 4.09 (s, 0.25 H), 3.87 (s, 3 H), 2.94 (t, *J* = 6.23 Hz, 2 H), 2.26 (t, *J* = 6.23 Hz, 2 H), 1.91 – 1.85 (m, 2 H), 1.74 – 1.68 (m, 2 H)

^13^C NMR (CDCl_3_, 100 MHz): δ= 160.35, 153.15, 152.28, 133.57, 128.60, 128.38, 128.02, 127.35, 126.23, 125.60, 125.52, 125.32, 124.54, 123.59, 117.57, 53.72, 32.80, 29.53, 27.39, 23.32, 23.10

HRMS (ESI+): *m*/*z*

[M + H]+ calcd for C_27_H_26_NO: 380.2014; found: 380.2054.

**
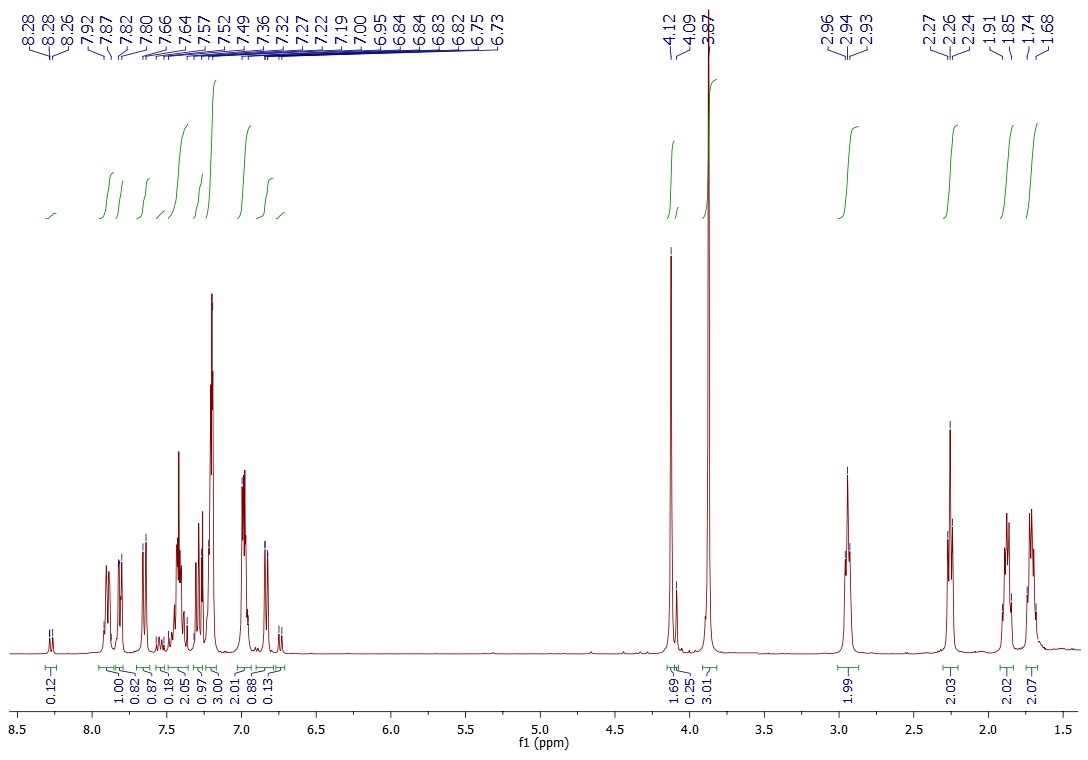
**

**Figure S33. ^1^H NMR of 2-methoxy-3-(1-naphthylmethyl)-4-phenyl-5,6,7,8-tetrahydroquinoline**


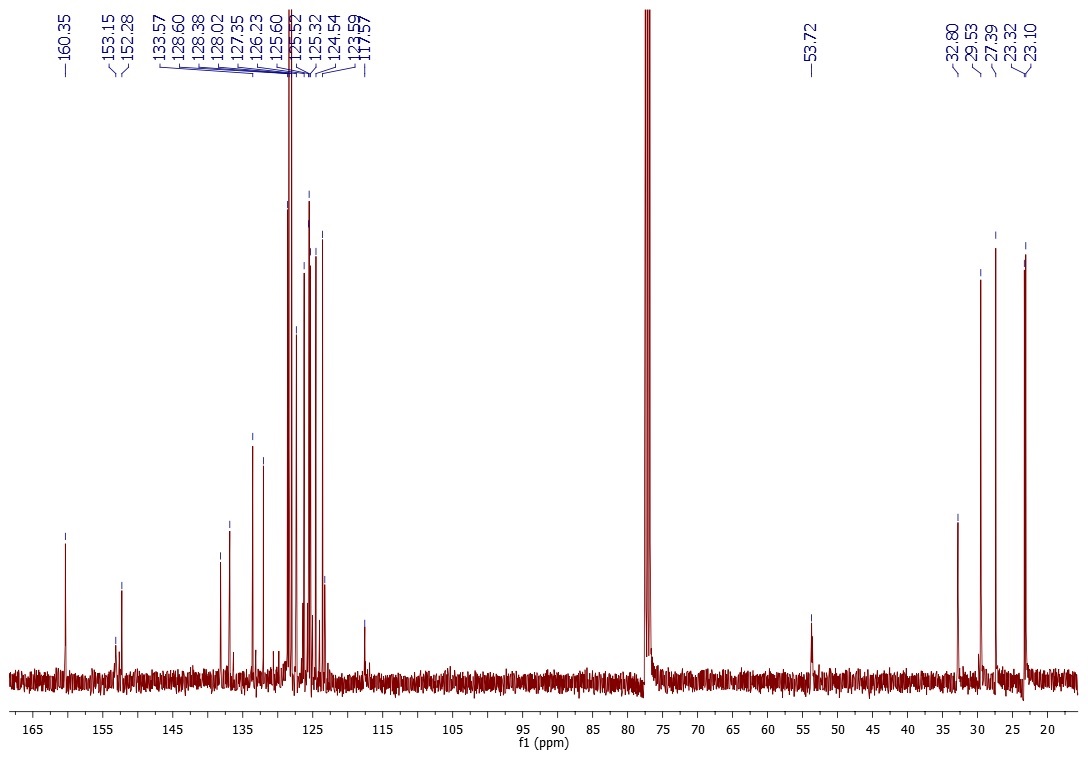


**Figure S34. ^13^C NMR of 2-methoxy-3-(1-naphthylmethyl)-4-phenyl-5,6,7,8-tetrahydroquinoline**

**BIOLOGICAL EVALUATION**


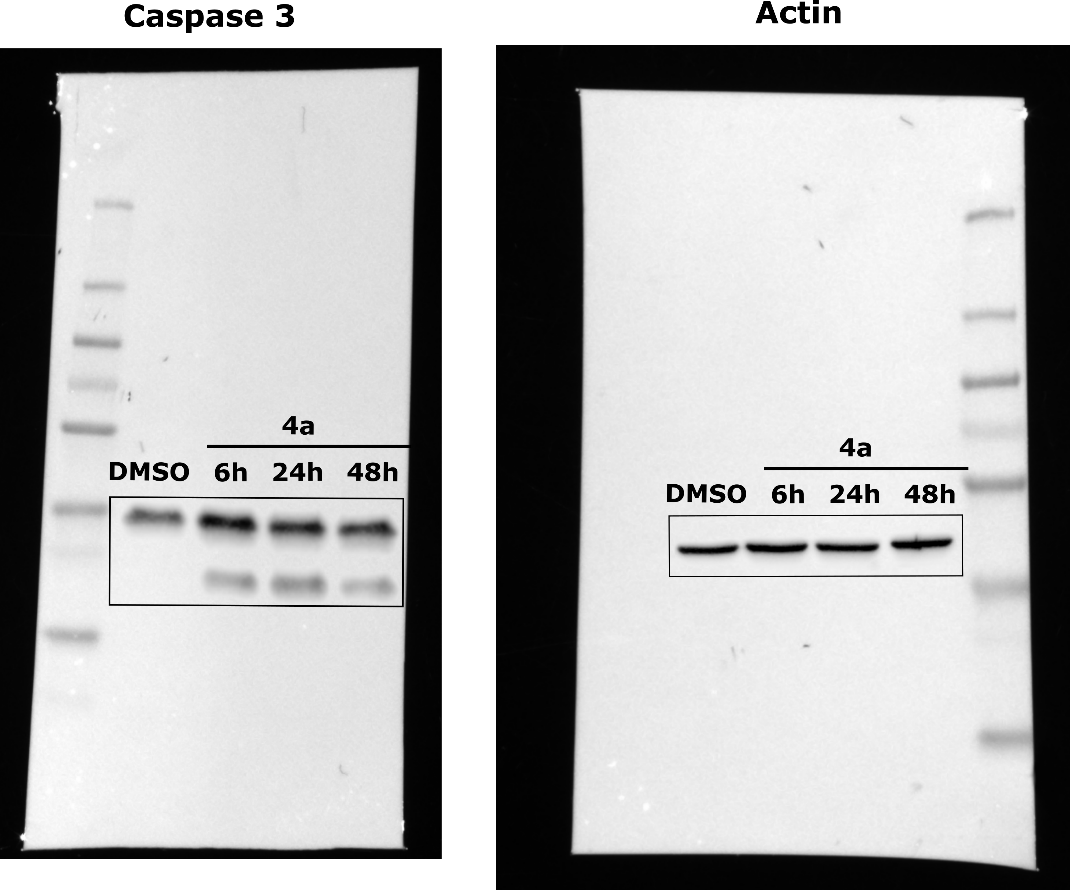


**Figure S35.** A full-length Western blot presented in **Figure 5** of the main article. Boxes denote the cropped regions of the blots.


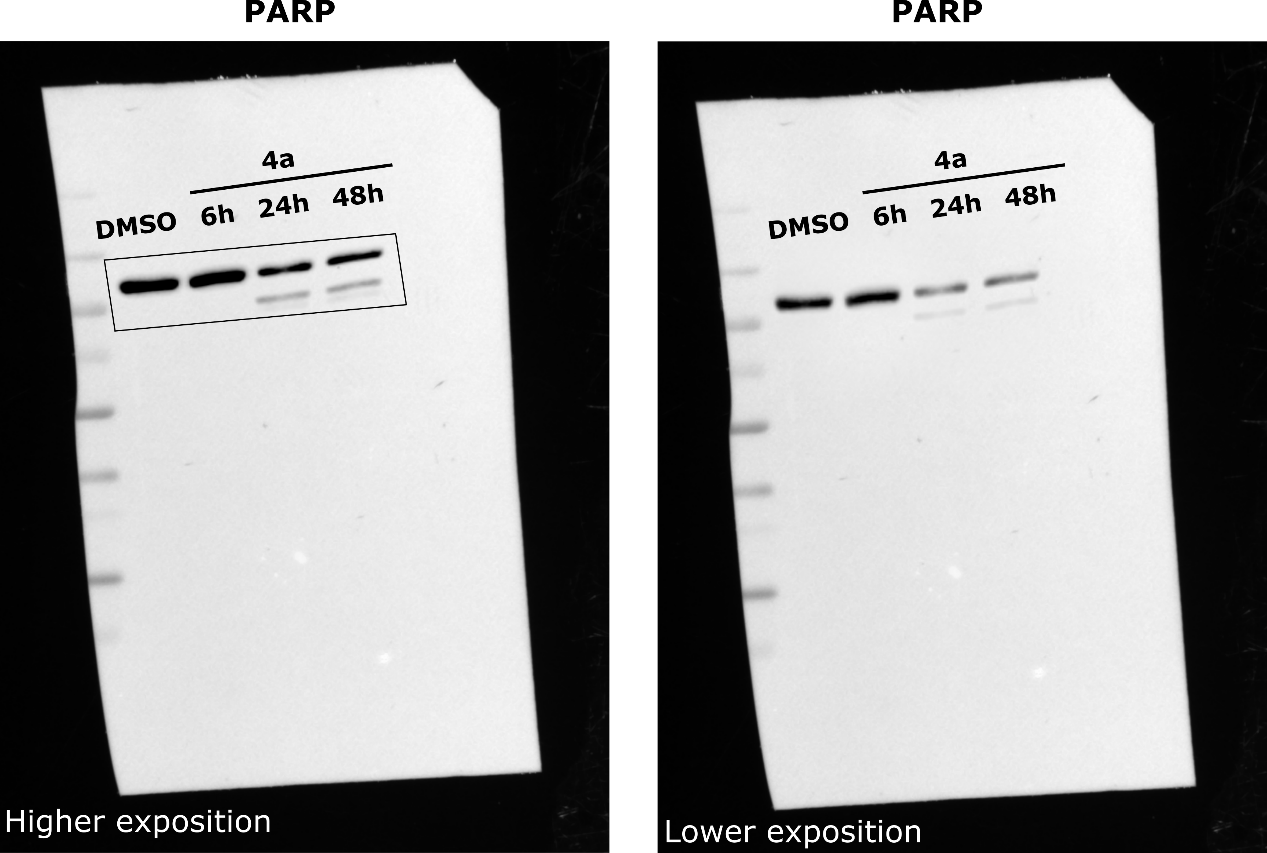


**Figure S36.** A full-length Western blot presented in **Figure 5** of the main article. Boxes denote the cropped regions of the blots.


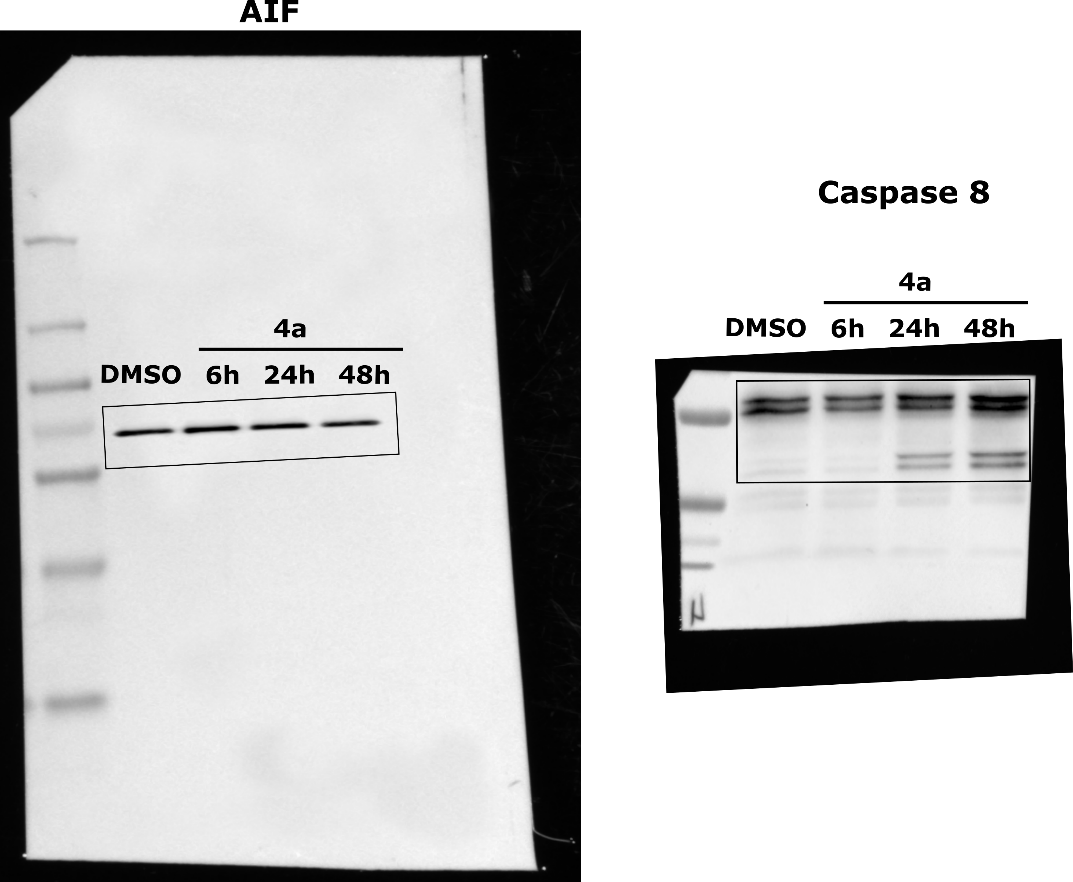


**Figure S37.** A full-length Western blot presented in **Figure 5** of the main article. Boxes denote the cropped regions of the blots.


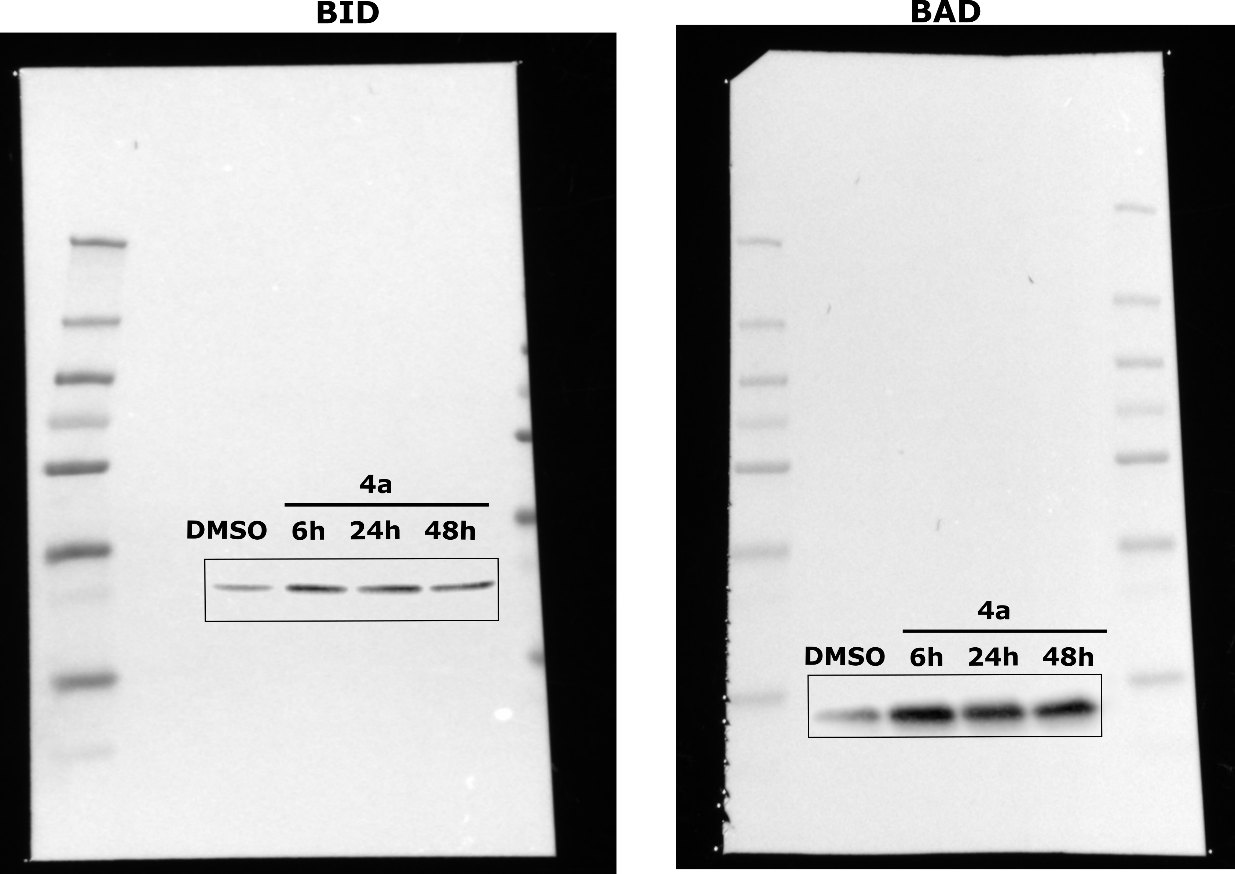


**Figure S38.** A full-length Western blot presented in **Figure 5** of the main article. Boxes denote the cropped regions of the blots.


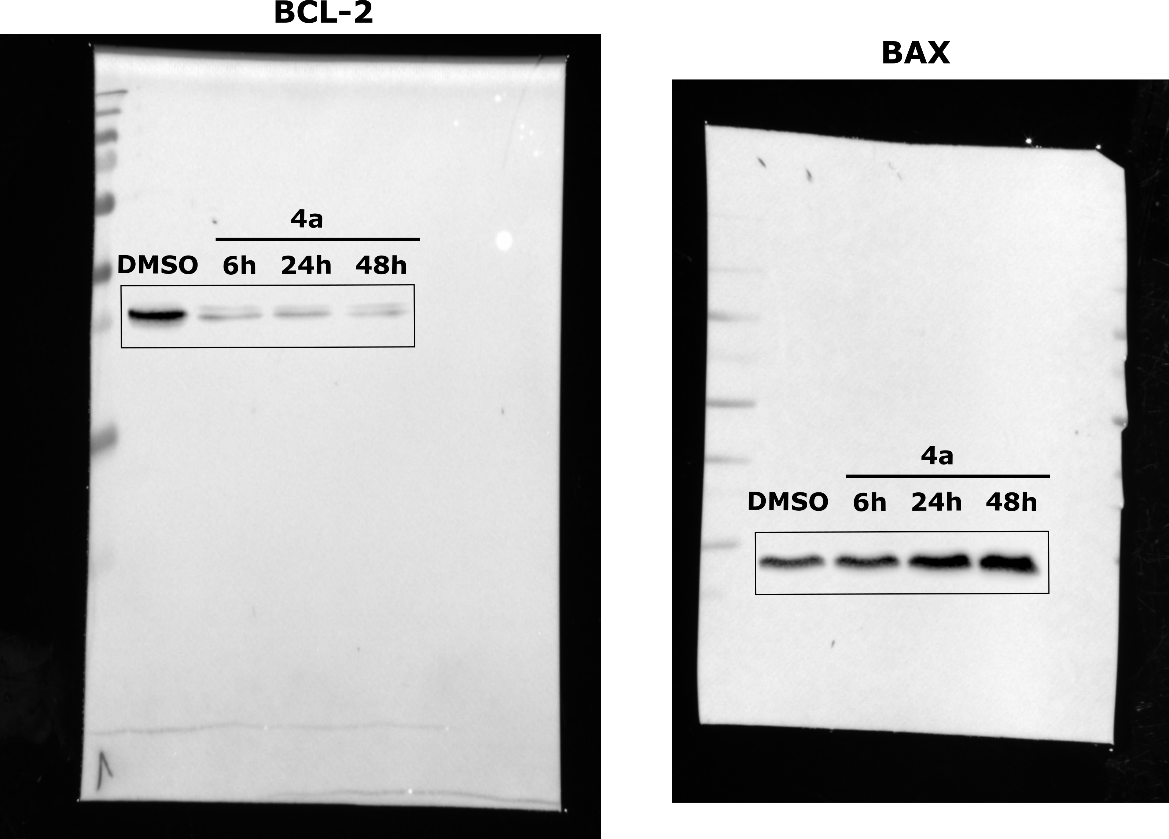


**Figure S39.** A full-length Western blot presented in **Figure 5** of the main article. Boxes denote the cropped regions of the blots.

**Figure S40
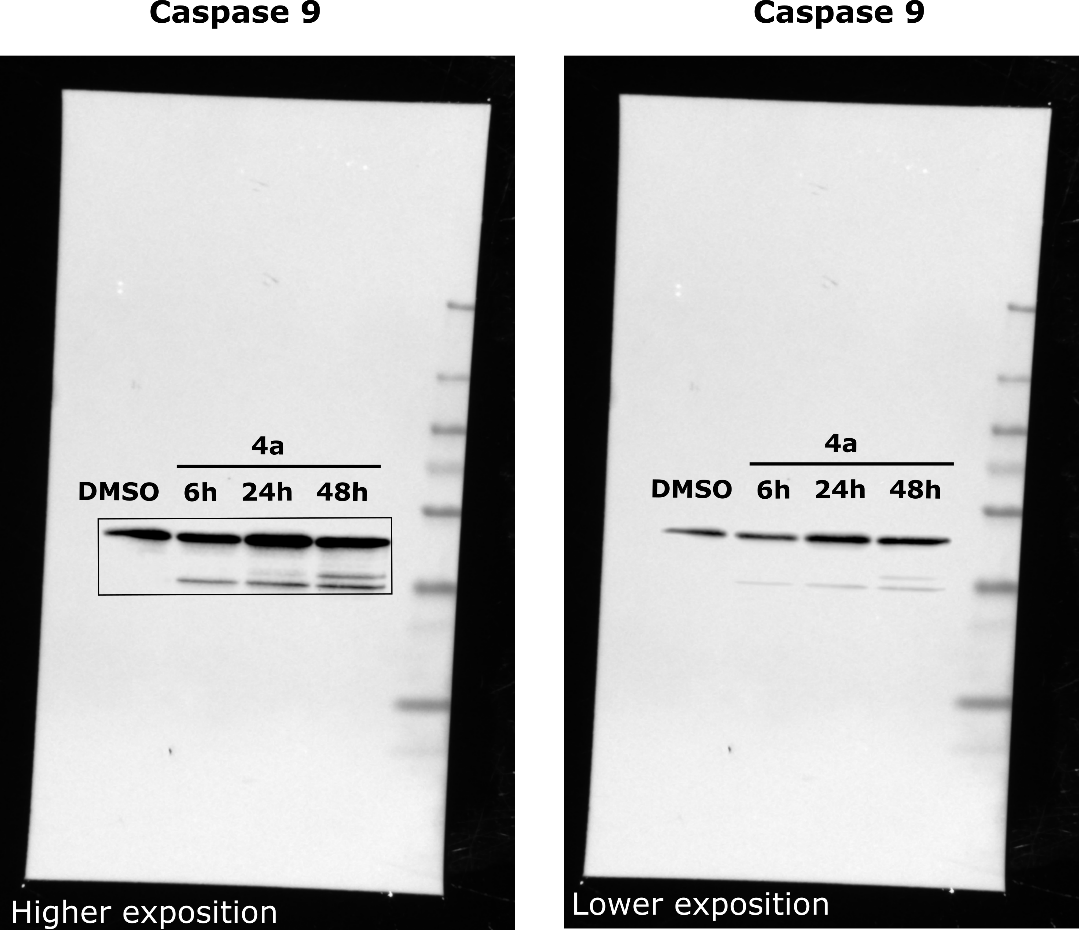
.** A full-length Western blot presented in **Figure 5** of the main article. Boxes denote the cropped regions of the blots.

**Table S1.** List of antibodies used in Western blotting

| Antibody name | Company | Dilution |
| --- | --- | --- |
| Anti-Caspase-3 (#9662) | Cell Signaling | 1:1000 |
| Anti-Caspase-9 (#9502) | Cell Signaling | 1:1000 |
| Anti-Caspase-8 (1C12) (#9746) | Cell Signaling | 1:1000 |
| Anti-PARP (#9542) | Cell Signaling | 1:1000 |
| Anti-BCL-2 (#4223) | Cell Signaling | 1:1000 |
| Anti-BID (#2002) | Cell Signaling | 1:1000 |
| Anti-BAD (ab32445) | Abcam | 1:1000 |
| Anti-AIF (#4642) | Cell Signaling | 1:1000 |
| Anti-BAX (#2772) | Cell Signaling | 1:1000 |
| Anti-actin (sc-1616) | Santa Cruz | 1:100 |
| Anti-mouse-HRP (715-035-150) | Jacson ImmunoResearch Labs | 1:10000 |
| Anti-rabbit-HRP (711-035-152) | Jacson ImmunoResearch Labs | 1:10000 |
| Anti-goat-HRP (705-036-147) | Jacson ImmunoResearch Labs | 1:10000 |
| Anti-mouse-HRP (715-035-150) | Jacson ImmunoResearch Labs | 1:10000 |
| Anti-rabbit-HRP (711-035-152) | Jacson ImmunoResearch Labs | 1:10000 |
| Anti-goat-HRP (705-036-147) | Jacson ImmunoResearch Labs | 1:10000 |

**REFERENCES**

1. Huilai Y., Jie M., Xuexi S., A method of preparing of blonanserin, CN 104447551 (2015).

2. Lyle M. P. A., Wilson P. T., Synthesis of a new chiral nonracemic C2-symmetric 2,2‘-bipyridyl ligand and its application in copper(I)-catalyzed enantioselective cyclopropanation reactions *Org. Lett*, **6**, 855-857 (2004).

3. Morel A. F., Larghi L. E., Selvero M. M., Mild, efficient and selective silver carbonate mediated O-alkylation of 4-hydroxy-2-quinolones: Synthesis of 2,4-dialkoxyquinolines, *Synlett,* **18**, 2755-2758 (2005).
